# Validation of Ultrasonic Harmonic Scalpel for Real-Time Tissue Identification Using Rapid Evaporative Ionization Mass Spectrometry

**DOI:** 10.1021/acs.analchem.1c00270

**Published:** 2021-03-31

**Authors:** Eftychios Manoli, Sam Mason, Lauren Ford, Afeez Adebesin, Zsolt Bodai, Ara Darzi, James Kinross, Zoltan Takats

**Affiliations:** † Department of Surgery and Cancer, Imperial College London, St Marys Hospital, London W2 1NY, United Kingdom; ‡ Department of Metabolism, Digestion and Reproduction, Imperial College London, South Kensington Campus, London SW7 2AZ, United Kingdom

## Abstract

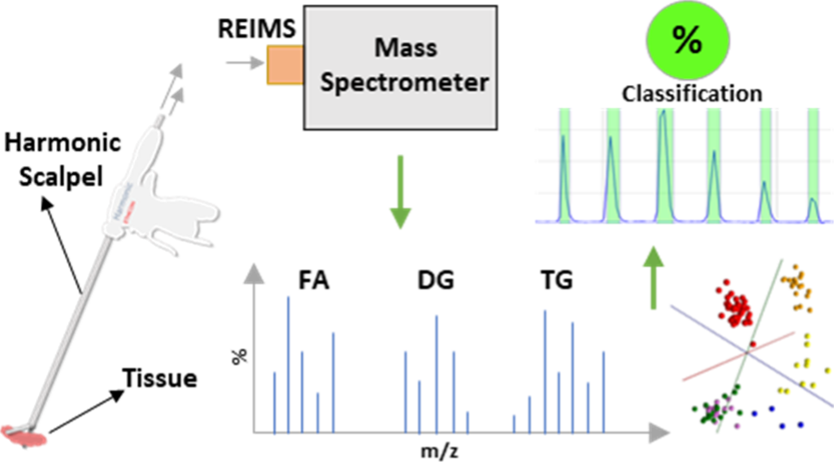

In this study, we integrate rapid evaporative ionization mass spectrometry (REIMS) with the Harmonic scalpel, an advanced laparoscopic surgical instrument that utilizes ultrasound energy to dissect and coagulate tissues. It provides unparalleled manipulation capability to surgeons and has superseded traditional electrosurgical tools particularly in abdominal surgery, but is yet to be validated with REIMS. The REIMS platform coupled with the Harmonic device was shown to produce tissue-specific lipid profiles through the analysis of porcine tissues in both negative and positive ionization modes. Comparison with other methods of electrosurgical dissection, such as monopolar electrosurgery and CO_2_ laser, showed spectral differences in the profile dependent on the energy device used. The Harmonic device demonstrated major spectral differences in the phospholipid region of *m*/*z* 600–1000 compared with the monopolar electrosurgical and CO_2_ laser-generated spectra. Within the Harmonic REIMS spectra, high intensities of diglycerides and triglycerides were observed. In contrast, monopolar electrosurgical and laser spectra demonstrated high abundances of glycerophospholipids. The Harmonic scalpel was able to differentiate between the liver, muscle, colon, and small intestine, demonstrating 100% diagnostic accuracy. The validation of the Harmonic device–mass spectrometry combination will allow the platform to be used safely and robustly for real-time *in vivo* surgical tissue identification in a variety of clinical applications.

## Introduction

**Figure 1:**
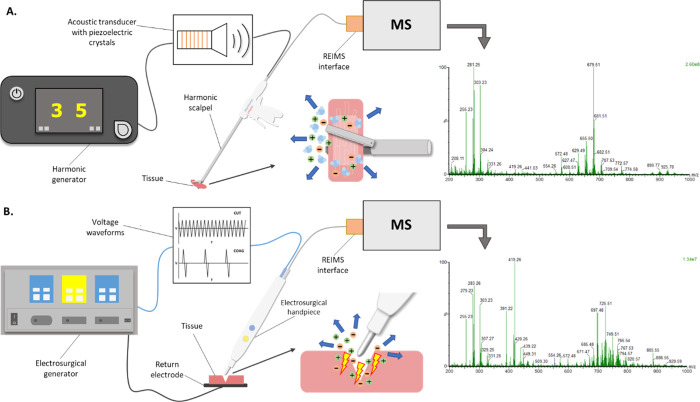
MS-guided surgery setup using the Harmonic scalpel (A) and monopolar electrosurgery (B). The mechanistic differences between the two instruments such as temperature and the amount and way the energy is transferred to the tissue result in spectral differences in spite of mechanistic similarities.

Precision surgery requires real-time analysis of tissue pathology to support intraoperative clinical decision making, safer surgery, and improved patient outcomes.^1^ However, there are few examples of this strategy in routine clinical use. Coupling surgical tools with ambient ionization mass spectrometry provide reproducible tissue and disease-specific data in real-time and does not require sample preparation.^2^ It is therefore a tangible example of how precision surgery could be deployed in practice. Rapid evaporative ionization mass spectrometry (REIMS) was developed for *in vivo* classification of human tissues through analysis of aerosols released during electrosurgical dissection^3,4^ and has demonstrated its ability in real-time cancer margin detection and tissue phenotyping. A major advantage of this platform is that it does not interfere with the standard surgical workflow, and it can be flexibly deployed across energy devices where rapid tissue ablation is used, yielding an aerosol. These energy devices include monopolar electrosurgical devices for open surgery^5^ and endoscopic applications,^6^ a bipolar handheld probe,^7,8^ a cavitron ultrasonic surgical aspiration instrument,^9^ and different surgical lasers.^10^ Alternative mass spectrometry (MS) techniques used intraoperatively for real-time diagnostics include MassSpecPen^11^ and SpiderMass.^12^ MassSpecPen performs a surface tissue extraction using water, and the extract is then transferred to the mass spectrometer for analysis using fluidics. This involves the risk of carryover and it is time-consuming; due to the use of water as an extraction solvent, this method also produces less-abundant mass spectra in tissues where there is high fat content such as normal breast tissue. The SpiderMass works in a similar way as REIMS, where analysis of biological tissue is done using a fibred IR laser, which causes resonant excitation of the endogenous water molecules which can be aspirated and transferred to the MS for analysis. Over the last two decades, there has been a progressive adoption of laparoscopic and minimally invasive approaches in general surgery. For example, in the UK, up to 76% of colorectal cancers are treated with this technique.^13^ The Harmonic scalpel is commonly used in abdominal minimally invasive surgery.^14,15^ The system is composed of a generator and a handpiece with an ultrasonic transducer, which converts electrical energy to high-frequency mechanical vibration (Figure 1). The mechanical vibrations are attenuated in the tissue and due to the heat generated by the dissipation of kinetic energy, the device simultaneously cuts and cauterizes tissues. The device operates at lower temperatures (<100 °C) compared to other surgical energy devices, and there is an overall reduction of lateral thermal tissue damage and spread.^16^ Subsequently, desiccation and charring effects on the tissues and blood vessels^17^ are much less pronounced compared to other electrosurgical instruments offering greater precision when the tissue is dissected. The process of dissection involves the formation of a largely aqueous aerosol which, similarly to electrosurgery, is chemically representative of the dissected tissue. Consequently, this aerosol can also be analyzed by MS using an REIMS-like approach, yielding another modality for MS-guided surgery. To date, REIMS has not been applied in the minimally invasive surgical setting and it is unclear if this technology is effective for this purpose, in part because the laparoscopic instrument is fundamentally different in both its form and method of energy deployment (Figure 1). In this prospective *ex vivo* preclinical study, we demonstrate the feasibility of coupling the Harmonic scalpel to the REIMS interface for minimally invasive surgery. This analysis compares the diagnostic performance of Harmonic to other methods of electrosurgical and laser dissection and defines the chemistry of droplet formation when using the Harmonic scalpel as a precision surgical device.

## Experimental Section

### MS Instrumentation

For the experiments described in this study, a Xevo G2-XS QToF mass spectrometer (Waters Corporation, UK) was used. The surgical aerosol produced using the different surgical instruments was transferred through a 3 mm internal diameter polytetrafluoroethylene (PTFE) tubing. As part of the setup, the Venturi air jet pump driven by medical air (1.8 bar) was used to transfer nascent surgical aerosol into the mass spectrometer. Vapors produced were coaspirated with a solvent solution of Propan-2-ol (Sigma-Aldrich, UK) for signal enhancement improving the quality of the data.^18^ Leucine Enkephalin (Sigma-Aldrich) (1 ng/μL) was used as a lock mass reference compound (*m*/*z* 554.2615 for the negative mode and *m*/*z* 556.2771 for the positive mode) with a flow rate set to 0.2 mL/min. Data were acquired in the negative and positive ion mode, in the *m*/*z* range of 100–1000. The sensitivity mode was used, with a scan time set to 1 s and data were collected in the continuous mode.

### Surgical Instruments

A commercially available surgical CO_2_ laser (Omniguide Surgical, MA, USA) with 10.6 μm wavelength was used. The laser was equipped with a 200 μm hollow flexible photonic band gap laser fiber (diameter 1.46 mm, length 1.8 m, Elevate Fibre, USA) which was connected to a BeamPath NEURO 10 cm straight handpiece for tissue ablation. The device was used in the superpulse mode, at a frequency of 330 Hz, a pulse off time of 3.3 ms, a pulse on time of 1–100 μs, and a peak power of 80 W. Helium gas (30 psi) was used to cool the fiber and avoid contamination during ablation. The laser power was set to 1–6 W. For electrosurgical experiments, a modified monopolar diathermy handpiece^19^ with a short straight blade (1 cm) was used, connected to a commercially available electrosurgery unit (Covidien, Medtronic, UK). Cut and coagulative modalities were set to 10–40 W. A commercially available 20 cm Harmonic ACE +7 (Ethicon Endo-surgery, part of Johnson and Johnson) was used for tissue analysis, with a tapered-tip blade and the power of the generator (GEN11, Ethicon Endo-Surgery, US) set to 1–5 W. The PTFE tubing for the smoke aspiration was attached to the shaft of the Harmonic scalpel with tape, with the end of the tube placed within 1 cm of the jaws (Figure S1).

### Laser Safety Regulations

Omniguide fibred CO_2_ laser falls into the class 4 safety category, so extra care was taken when the laser was in use. Our laboratory is equipped with a laser interlock control system (Lasermet Ltd) with LED laser warning signs. The system follows all the European Safety Requirements and Standards [(EN ISO 13849–1 PLe (Cat 4 safety system, Performance Level “e”), EN61508 (SIL 4), EN61010, and EN60947-1)]. The laboratory is used by authorized users only who follow the local rules and had appropriate training. Appropriate safety glasses (190–398 nm + 9000–11,000 nm OD7+, 93% VLT EC2) were used during the experiments.

### Aerosol Inhalation Safety Regulations

Appropriate breathing masks and personal protective equipment were used during all the *ex vivo* analysis. A fume extractor (Wellar WFE 2ES) with a funnel nozzle kit, along with a smoke evacuator and venting system attached to the Venturi housing (Covidien RapidVac equipped with an ULPA filter), was used during all the experiments.

### Samples

Frozen (−20 °C) porcine organs (liver, colon, muscle, and small intestine) were acquired from Fresh Tissue Supplies (East Sussex, UK). The samples were left to thaw at room temperature, and multiple analysis points were taken using the different surgical tools.

### Statistical Analysis and Spectra Interpretation

Raw spectral data were processed in Abstract Model Builder (AMX) (v.1.0.2055.0, Waters Research Centre, Hungary), having undergone background subtraction, normalization, and lock mass correction using Leucine Enkephalin (at *m*/*z* 554.2615 for the negative mode and *m*/*z* 556.2771 for the positive mode). For spectral processing, background subtraction was performed using a locally adaptive model of noise in MassLynx (v.4.2), which adjusts the zero level in the continuum spectrum to lessen the effect of chemical noise. The data points were generated using one spectrum per analysis point. The data were imported into SIMCA (v. 16.1, Umetrics, Sweden) where multivariate statistical analysis was performed, including principal component analysis (PCA) and orthogonal partial least squares discriminant analysis (OPLS-DA). Bins between the *m*/*z* of 550–600 were excluded from the analysis to avoid any lock mass interferences. Univariate statistical analysis and box plots were made in R Studio (v1.1.419) and loading plots were made using AMX.

### Ultraperformance Liquid Chromatography–MS

A sample preparation procedure was adopted using protein precipitation at 4 °C (1:5,Water/IPA).^20,21^ A 13 min gradient elution profile was used on an ultraperformance liquid chromatography (UPLC) binary solvent manager. The mobile phase A consisted of water/isopropanol:acetonitrile (2:1:1), 5 mM ammonium acetate, 0.05% acetic acid, and 20μM phosphoric acid. The mobile phase B consisted of isopropanol/acetonitrile (1:1), 5 mM ammonium acetate, and 0.05% acetic acid. The gradient profile was 99% A (0.0–2.0 min), 70% A (2.0–11.5 min), 10% A (11.5–12.0 min), 0.1% A (12.0–12.50 min), 35% A (12.50–12.55 min), 70% A (12.55–12.65 min), 99% A (12.65–12.75 min), and 99% A (12.75–13.25 min). The flow rate was set to 0.6 mL/min. An Acquity UPLC BEH C8, 2.1 × 100 mm, 1.7 μm column (Waters Corporation, USA) was used for chromatographic separation and it was maintained at 55 °C. Electrospray ionization–time-of-flight–MS was used for detection in the positive and negative mode (Xevo G2-S QTof, Waters, UK). Both MS and MS/MS data scans were acquired for 0.1 s in the centroid mode. In the positive mode, MS conditions were as follows: cone voltage 25 V, capillary voltage 2 kV, source temperature 120 °C, desolvation temperature 600 °C, and desolvation gas 1000 L/h. In the negative mode, MS conditions were as follows: cone voltage 25 V, capillary voltage 1.5 kV, source temperature 120 °C, desolvation temperature 600 °C, and desolvation gas 1000 L/h. Acquisition was performed from *m*/*z* 50 to 2000.

## Results and Discussion

Preliminary experiments were performed to assess the feasibility of using the Harmonic scalpel as a surgical aerosol source for REIMS and to compare the resulting spectral information with other instruments for surgical dissection and to assess the ability to create differential mass spectral fingerprints representative of tissue type when using the Harmonic scalpel. It was expected that the lower temperature, along with the different mechanism of tissue ablation would lead to differences in the resultant spectra when using the Harmonic scalpel when compared to CO_2_ laser and diathermy.

### Generator Power Settings

**Figure 2:**
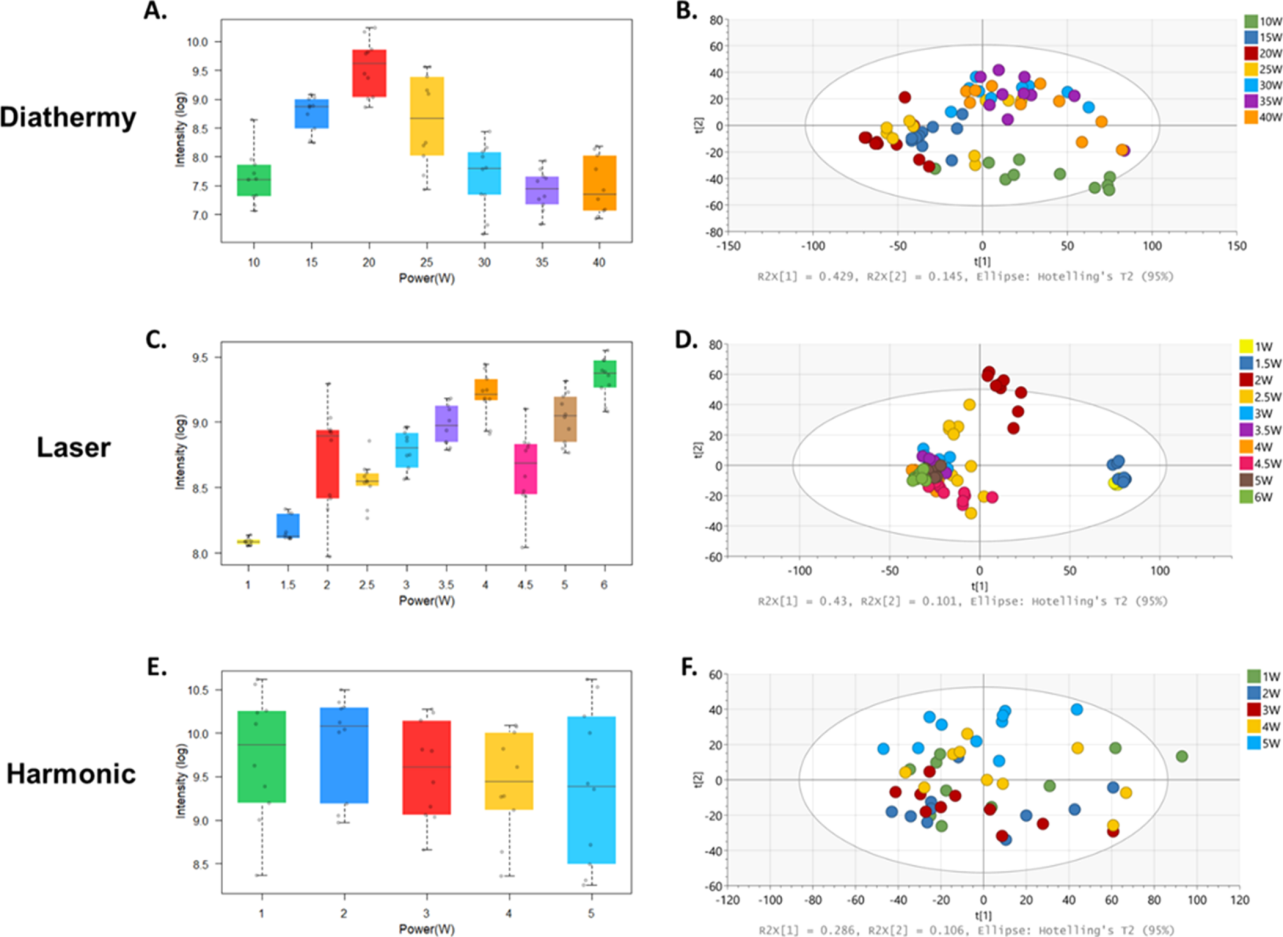
(A,C,E) Box plots showing the log scale intensity of the 20 most intense ions in the *m*/*z* range of 600–1000, for individual power settings for each surgical instrument. The box represents the interquartile range with the median shown. The whiskers represent the range of data points, excluding outliers, which were defined as five times the standard deviation from the median value. Additionally, raw data are represented using jitter points. Raw data signal intensities (with no log scales) including minimum and maximum values for diathermy, laser, and harmonic were 787.8–27,923.8, 2911.9–14,049.5, and 3855.5–40,751.8, respectively. For spectral processing, background subtraction and lock mass correction using leucine enkephalin (*m*/*z* 554.2615) was applied. (B,D,F) PCA plots of the different power settings using the different surgical instruments in the *m*/*z* range of 600–1000. Ten distinct sampling points were made sequentially for each individual power setting and for each instrument during the same analytical session.

Previous groups made recommendations for power settings for different surgical tools;^5,22^ however, this is likely to be specific to the tissue type analyzed with its specific metabolic composition. It was therefore deemed necessary to assess the optimal power settings for the three energy devices (monopolar diathermy, CO_2_ laser, and the Harmonic device) in parallel using a porcine liver model. Porcine liver was used because of the high-water content and the low mechanical resistance, making it suitable for experimental *ex vivo* studies, producing rich REIMS signals.^10^ Ten distinct sampling points were made sequentially for each individual power setting and for each instrument during the same analytical session. The negative ionization mode was used, as REIMS spectra using electrosurgery have shown to produce superior signals in the negative mode.^23^ The signal intensities based on the 20 most intense peaks in the 600–1000 *m*/*z* lipid range for all the power settings for all three instruments can be visualized using box plots (Figure 2). PCA was also performed to identify any patterns in the data (Figure 2). For monopolar diathermy, power settings were tested starting from 10 W up to 40 W, with 5 W increments. Data between 15 and 25 W appeared to have the higher signal intensities compared to the other power settings (Figure 2A) and they clustered together on the PCA plot (Figure 2B). An optimized heating power of 20 W was chosen, as it showed significantly higher signal intensity compared to the other settings. For the CO_2_ laser, higher signal intensity was associated with increased power settings (Figure 2C). Similar signal intensities were observed at 3, 3.5, 4, 5, and 6 W, showing less variability and clustering together on the PCA plot (Figure 2D). This main cluster of data is representative of the laser when producing high signal intensities in the *m*/*z* range of 600–1000. Lower power settings at 1 and 1.5 W showed the lowest signal intensities and were clustered far from the main cohort (Figure 2D) and thus considered partial outliers, demonstrating significant spectral variation compared to increased powers. It was observed by the operator that with the power settings at 1, 1.5, and 2 W, it was not high enough to deliver radiative heat and ablation to the tissue; thus, no biological signal was observed and the signal is solely based on background noise peaks. A reduction in the signal-to-noise ratio at 4.5 W was observed due to the elevated noise level. An optimum heating power at 3 W was chosen, considering the time of the analysis (longer with lower power settings), the thermal spread, and damage of the tissue that was observed with the higher power settings. With the harmonic device, similar signal intensities were observed across the range of the settings (Figure 2E), and the PCA plot showed high variability with no clear patterns or clusters observed (Figure 2F). While little spectral variation was present across different powers, the power setting of 5 W was chosen, as it was reported from other studies that with increasing power setting, there is a reduction in the dissection/analysis time.^24^ The common generator settings for the surgical use of the harmonic device are normally set between 3 W and 5 W, with the 3 W set as a minimum and 5 W as a maximum. The setting at 3 W is considered to be more coagulative for vessel sealing compared to the maximum 5 W that has greater cutting efficiency.^25^

### Surgical Tool Comparison

#### Negative Mode

**Figure 3:**
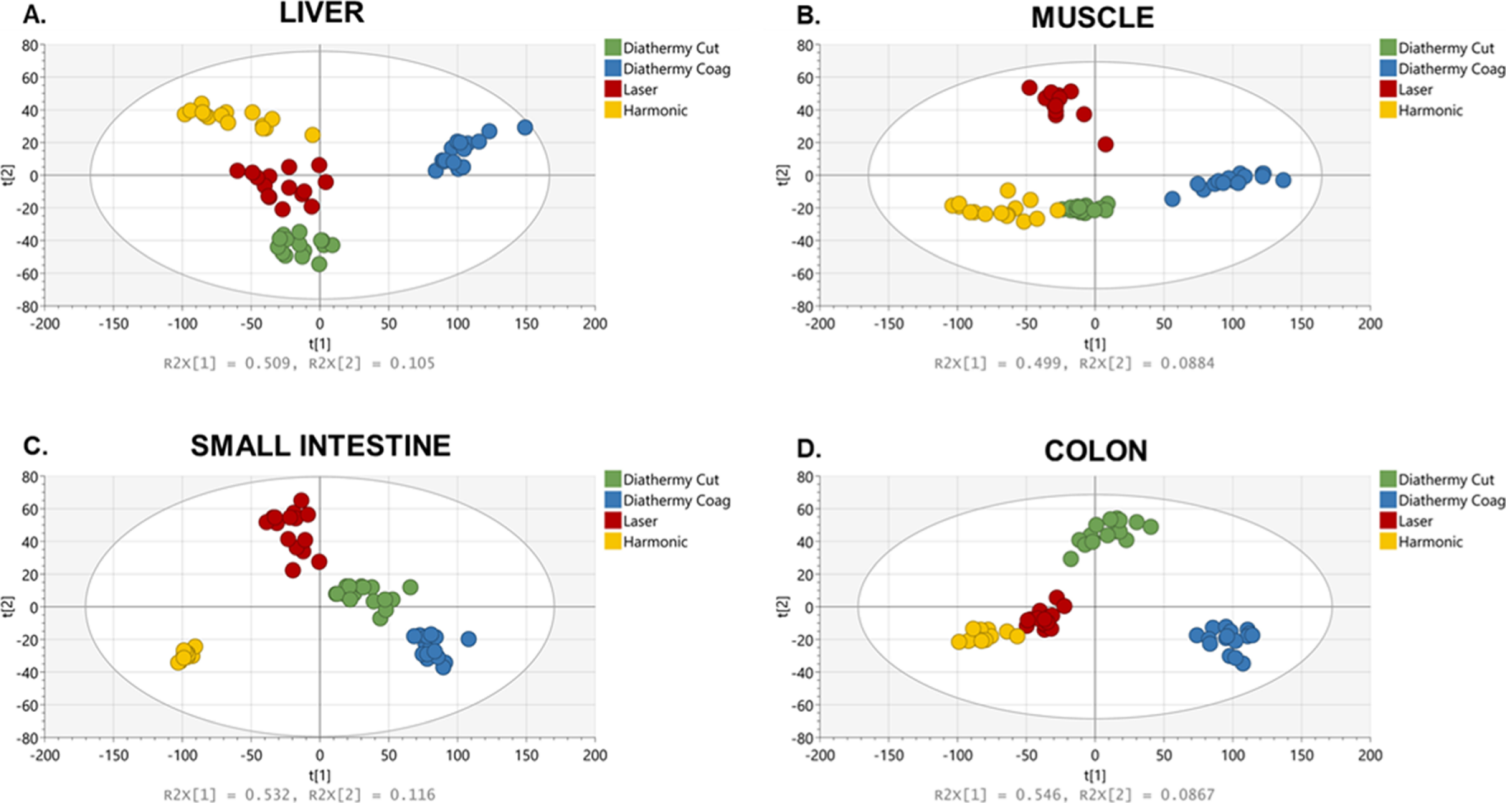
PCA plots using different surgical tools on (A) liver tissue, (B) muscle, (C) small intestine mucosa, and (D) colon mucosa within the *m*/*z* range of 100–1000. The tolerance ellipse of the two-dimensional score plot was based on Hotelling’s T2 with a significant level set to 0.05. For each surgical tool, a homogeneous tissue sample was analyzed sequentially in 15 locations. All measurements were conducted in the same analytical session.

The Harmonic scalpel, monopolar diathermy, and CO_2_ laser were tested on porcine liver, muscle, colon mucosa, and small intestine mucosa (Supporting Information—Table S1). For each instrument, a dedicated and homogeneous tissue sample was analyzed consecutively across 15 locations. All analysis points were conducted across a single analytical session. PCA plots were created to visualize the data and identify any clustering trends and patterns (Figure 3). The PCA of the data obtained using the different surgical instruments on various tissue types in the negative mode in the *m*/*z* range of 100–1000 shows distinct clustering, with low intragroup variance compared to intergroup variance, which implies that the main source of variation is the type of instrument being used (Figure 3).

**Figure 4:**
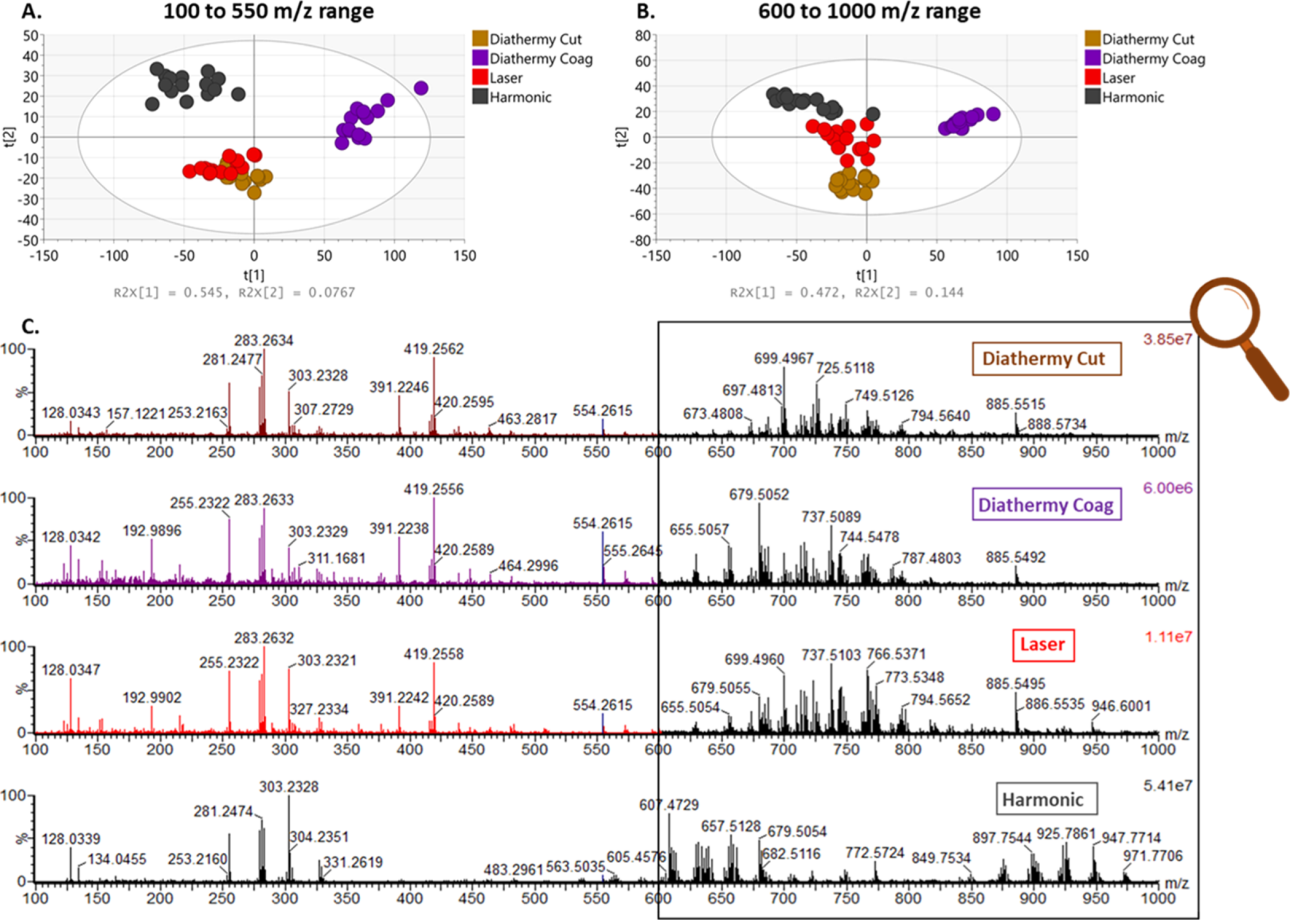
PCA plots obtained by the analysis of data using different surgical instruments on the porcine liver sample in the *m*/*z* range of 150–550 (A) and 600–1000 (B) in the negative mode, showing a separation between the Harmonic scalpel and the other instruments. Each point represents an analysis event. The tolerance ellipse of the two-dimensional score plots was based on Hotelling’s T2 with a significant level set to 0.05. (C) Comparison of averaged MS spectra acquired on liver tissue in the negative mode using a Xevo S2-XS instrument (Waters Corporation, UK). The magnified lipid area (*m*/*z* range of 600–1000) shows significant spectra differences between the different tools.

Spectral comparison was made across the *m*/*z* range of 100–1000 for all the different instruments on all tissue types (Supporting Information—Figure S2). Five background-subtracted spectra were averaged for each data point. In the case of pork liver tissue, there was good separation of the data acquired by the Harmonic scalpel compared to the other tools in the *m*/*z* ranges of 100–550 and 600–1000 (Figure 4A,B).The Harmonic data clustering together, separated from the other tissue classes, suggests marked spectral differences, something that can be easily observed on the corresponding spectra (Figure 4C). The first two components were accountable for most of the variation in the data set with PC1 explaining 54.5% of the variance followed by PC2 of 7.6% in the *m*/*z* range of 100–550. Similar variation was observed in the *m*/*z* range of 600–1000 showing PC1 47.2% variance and PC2 14.4%. The significant variance between the different groups across the whole mass range can be also seen by looking at the principal component loadings of the first two components (Supporting Information—Figure S3). In addition to multivariate statistical analysis, univariate analysis was also performed to identify the statistically significant ions responsible for the separation between the different surgical tools. The ions identified to be statistically significant include fatty acids, glycerophospholipids, and glycerolipids (Supporting Information—Figures S4 and S6). Fatty acids of relatively high abundance were observed in the *m*/*z* range of 100–550 for all spectra and were putatively identified using Lipid Maps database^26^ (Supporting Information—Table Matrix 1). It was found that fatty acids such as the palmitic acid (16:0) at *m*/*z* 255.2324, the oleic acid (18:1) at *m*/*z* 281.2477, the stearic acid (18:0) at *m*/*z* 283.2631, and the arachidonic acid (20:4) at *m*/*z* 303.2328 were among the most statistically significant (*p* < 0.05) fatty acids observed, comparing differences in relative abundance between devices. Comparing the fatty acid intensities for each instrument mentioned above, the Harmonic shows not only higher intensity fatty acid ions but also the greatest variation of the distributed data when compared to the other instruments (Supporting Information—Figure S4). In addition, the data from monopolar electrosurgery in the coagulative mode had the lowest relative abundance of fatty acids and much noisier spectra, something that can be possibly explained by the coagulation effect on the tissue using modulated high voltage that only affects the superficial layers of the tissue. The energy in this case causes heating reaching very high temperatures going beyond 200 °C where carbonization occurs. In addition, significant spectral differences were observed in the *m*/*z* range of 600–1000, where completely different ion patterns were seen between the Harmonic device and the other surgical tools. In the *m*/*z* range of 600–800, very low-intensity glycerophospholipid ions were observed in the case of the Harmonic scalpel compared to the other devices (Supporting Information—Figure S5A). There was an absence of distinctive phospholipids such as *m*/*z* 697.4812 [PA(36:3) – H]^−^, 723.4965 [PA(38:4) – H]^−^, 744.5513 [PE(18:1_18:0) – H]^−^, 747.5109 [PG(18:1_16:0) – H]^−^, and [PI(20:4_18:0) – H]^−^ at *m*/*z* 885.55 and instead remarkably high-intensity triglyceride patterns were detected (Supporting Information—Figure S5B). Moreover, significantly higher intensity diglyceride ions such as *m*/*z* 629.4890 [DG(34:1) + Cl]^−^ and 655.5053 [DG(36:2) + Cl]^−^ were observed and putatively identified using online databases (Lipid Maps and Metlin^27^). The relative abundance of these ions was significantly higher in the Harmonic spectra, as well as the variation within, compared to the laser ablation and the diathermy data as seen in the box plots in the Supporting Information—Figure S6. The actual ion formation mechanism is based on the rapid thermal evaporation of aerosol particles in the atmospheric interface as seen by electrosurgical and laser surgical tools combined with REIMS. However, due to the lower temperature and differing mechanism (dissipation of mechanical vibration kinetic energy as opposed to joule heating) of tissue ablation, droplet formation and subsequent mass spectra representing the tissue are expected to display different trends. The spectra generated from the aerosol of the Harmonic scalpel also differed from the other surgical tools through the absence of ions within the *m*/*z* range of 350–500 such as *m*/*z* 391.22, 415.22, 417.23, and 419.25 (Figure 3C). Since these ions are fragments (neutral losses of fatty acid chains) of major deprotonated glycerophospholipid species observed within the range of *m*/*z* 600–1000 in the laser and diathermy data, it is in agreement with the absence of common phospholipid ions.

**Figure 5:**
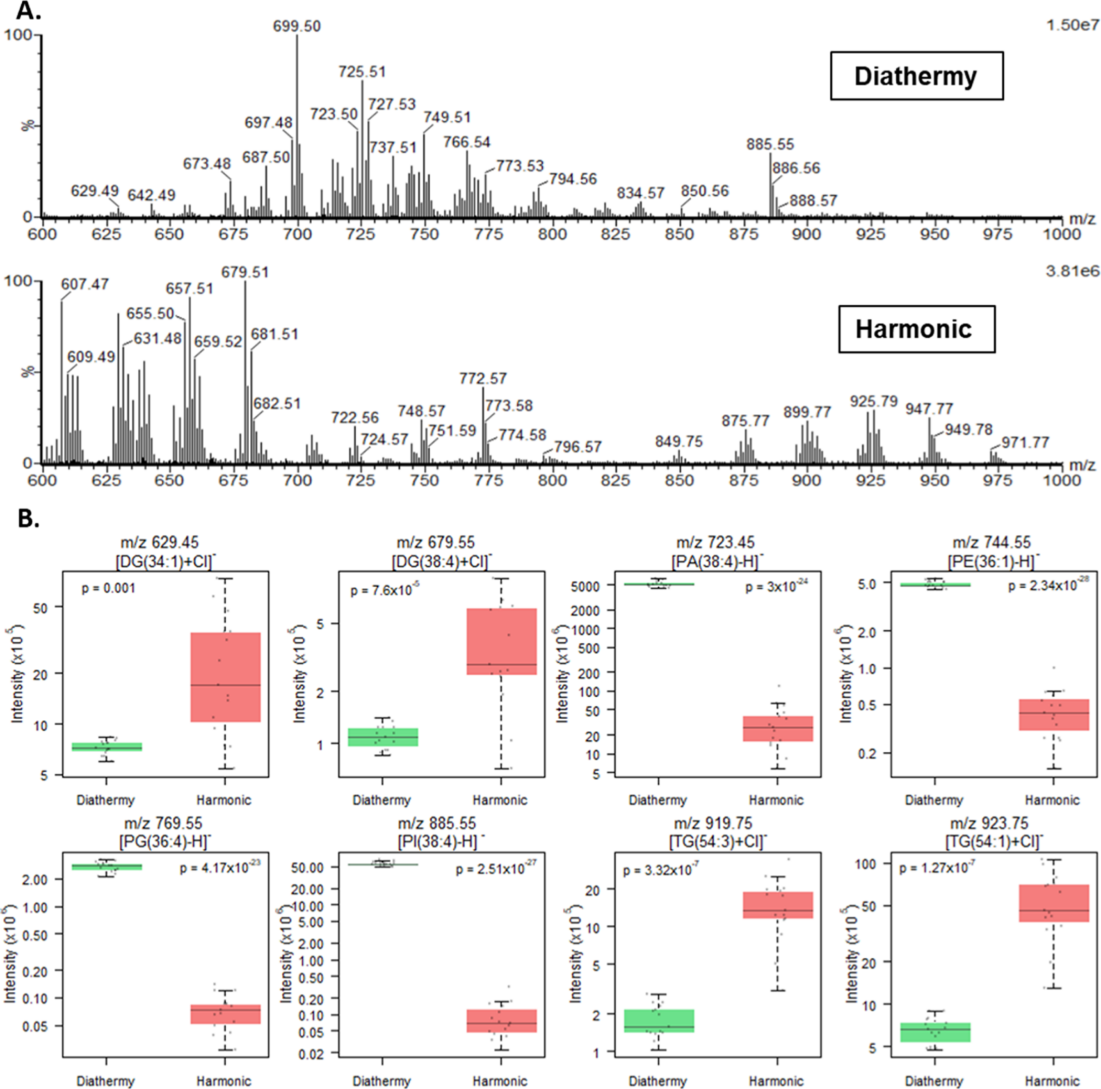
(A) REIMS spectra comparison of the diathermy and the Harmonic data on porcine liver in the *m*/*z* range of 600–1000. High-intensity phospholipids were seen in the diathermy data compared to the Harmonic spectra where high-intensity diglycerides and triglycerides were seen. (B) Box plots showing some of the lipids that were of statistically significantly different relative abundance between the Harmonic and diathermy surgical tools, using a log scale of intensity. The box represents the interquartile range with the median shown. The whiskers represent the range of data points, excluding outliers, which were defined as five times the standard deviation from the median value. Additionally, raw data are represented using jitter points.

**Figure 6:**
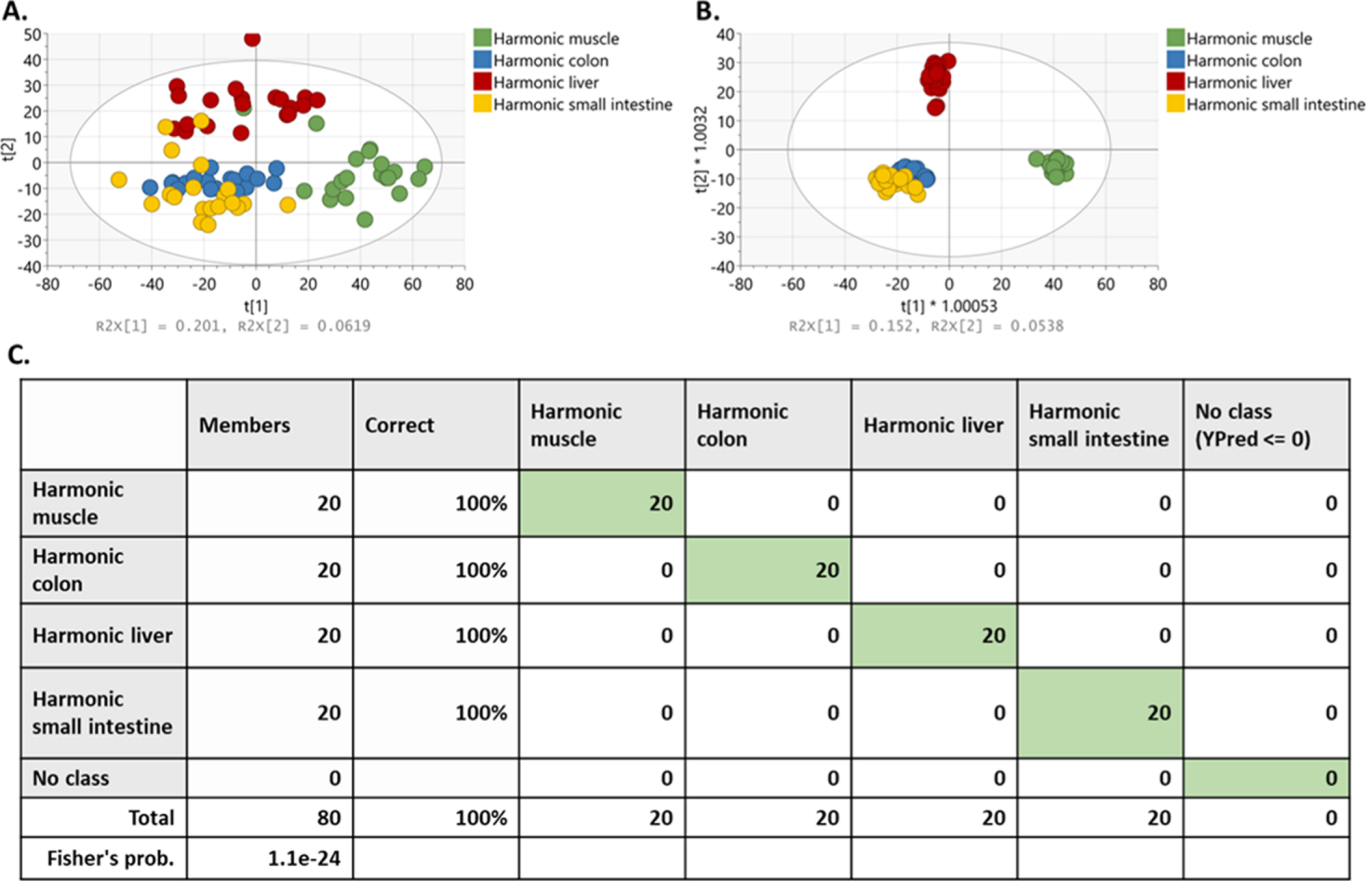
Multivariate statistical models of different porcine tissue types (liver, muscle, colon, and small intestine) in the *m*/*z* range of 600–1000 in the negative mode using the Harmonic device. A good separation between the different tissue types was observed in (A) PCA score plot with PC1 = 20.1% and PC2 = 6.19% and (B) OPLS-DA with *R*^2^*Y* = 0.965 and *Q*2 = 0.87. (C) Overall diagnostic accuracy of 100% was observed using leave-one-spectrum-out cross-validation.

The significant spectral differences observed between the diathermy and the Harmonic data, especially observed in the phospholipid range (Figure 5A) can be explained by the fact that the Harmonic device does not destroy cellular structures; hence, most of the aerosol is formed of interstitial fluid and will contain mostly non-membrane-forming lipids (Figure 5B). With the Harmonic device, we not only observed a three times higher total ion count (TIC) compared to the diathermy but also observed a longer duration of sampling compared to the diathermy (mean TIC: Harmonic 8.29 × 10^8^*vs* diathermy 2.87 × 10^8^, sampling duration range: Harmonic 8–10 s *vs* diathermy 3-4 s). We observed higher variability in signal intensity between the Harmonic sampling points, but this is not accounted for the differences we have seen in sampling duration. Although there are fundamental differences between the two devices on how the tissue is manipulated, the tissue surface contact, the activation times, the dispersion of aerosol generated, and how the energy is distributed within the tissue with a diathermy electrode and the Harmonic jaws, we are unable to explain at this point the reason for high variation observed between the Harmonic sampling points. A list of the ions whose difference in relative abundance has the highest statistical significance between the diathermy and the Harmonic REIMS spectra can be found in the Supporting Information—Matrix11. To reveal the identity of some of the significant features, samples of the same porcine tissue were extracted and analyzed by LC–MS/MS (Supporting Information—Figure S7). In some cases, more than one possible lipid structure was identified based on the same extracted lipid mass (with different retention times), indicating a mixture of two or more lipids identified under the same parent ion (Supporting Information—Figure S8). Supervised multivariate statistical analysis (OPLS-DA) was also performed using the data obtained using a Harmonic device and different porcine tissue types (porcine liver, muscle, colon, and small intestine) in the negative mode. The *m*/*z* range of 600–1000 was used, where intact structural or storage lipids lipid metabolites can be utilized for tissue identification in a robust manner, that is, the contribution of background is minimal. PCA showed some identifiable clusters and the greatest source of variation appeared to be the tissue type analyzed using the Harmonic device. Based on the ellipse Hotelling’s T2, a single sampling point was observed as an outlier (Figure 6A). Clear separation by tissue type was observed in the OPLS-DA model, showing high predictive ability (*R*^2^*Y* = 0.965 and *Q*^2^ = 0.87) (Figure 6B). The ability of REIMS to distinguish different porcine tissue types was assessed using leave-one-spectrum-out cross-validation, showing an overall identification accuracy of 100% (Figure 6C).

#### Positive Mode

**Figure 7:**
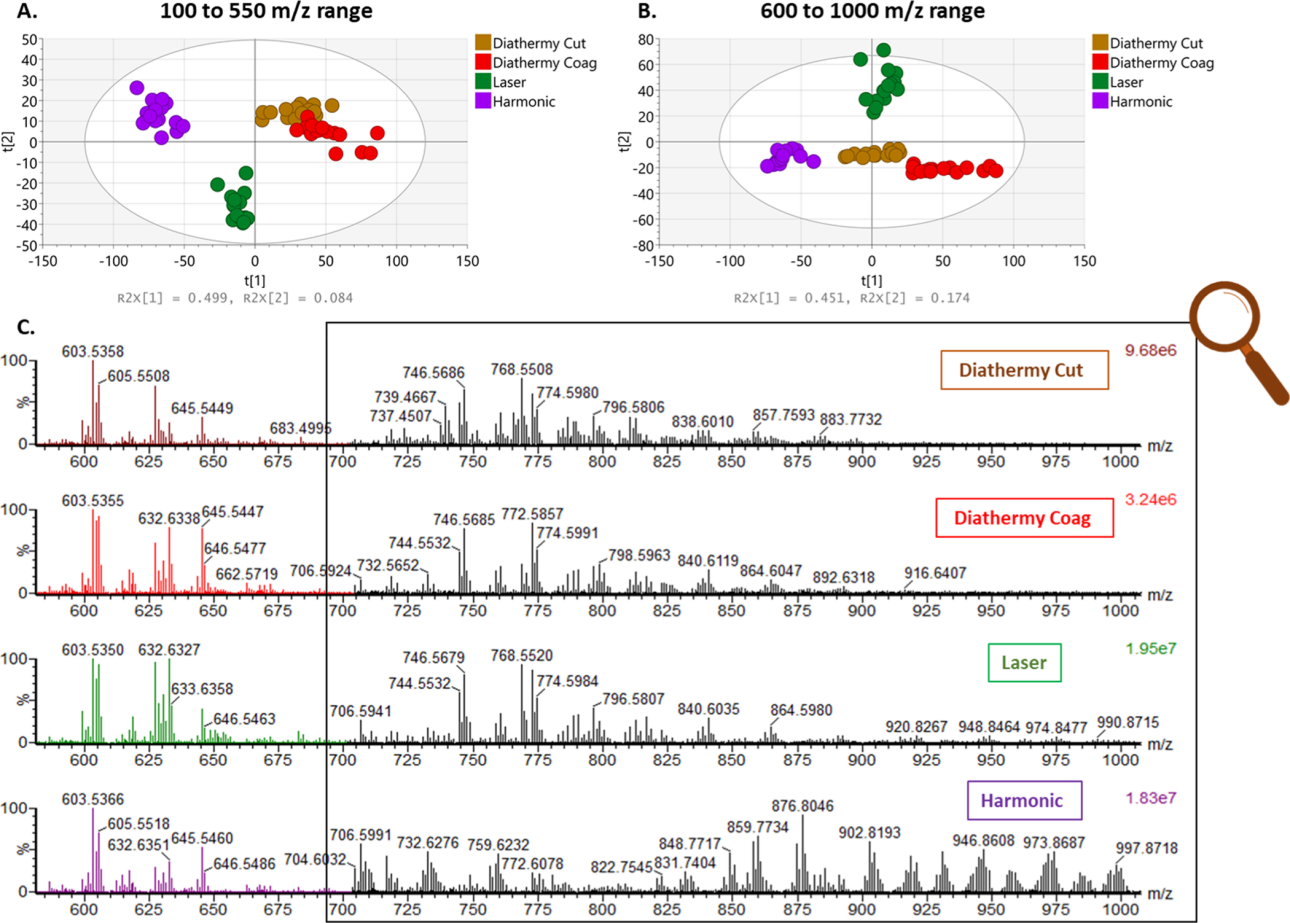
(A) PCA of data obtained using different surgical tools on porcine liver in the *m*/*z* range of (B) 600–1000 and (B)100–550 in the positive mode, showing a good separation between the Harmonic scalpel and the other instruments. Each point represents a sampling point. (C) Comparison of averaged MS spectra acquired on liver tissue in the positive mode using a Xevo S2-XS instrument (Waters Corporation, UK). Distinctive spectral differences were observed (as they were seen in the negative mode) especially in the range of 800–1000, where high-intensity triglycerides were seen in the Harmonic spectra.

There is a variety of lipids classes in biological tissues that are more likely to be seen in the positive mode, especially when these are associated with a specific type of disease.^28^ These metabolic components are more likely to form positively charged ions than negatively charged ions and they could give a further insight into tissue biology. Thus, experiments were also carried out using the different surgical tools on pork liver in the positive ion mode. PCA in the *m*/*z* range of 100–550 (Figure 7A) shows a cluster separation, with the first two principal components being responsible for most of the separation. As seen in the PC1 loading plot (Supporting Information—Figure S9A), the separation is caused by the *m*/*z* 121.0294 which appears to be dominant in all the spectra and is yet to be identified. In the *m*/*z* range of 600–1000, good separation was observed between the surgical instruments, as seen in the PCA plot with PC1 = 45.1% and PC2 = 17.4% (Figure 7B and Supporting Information—Figure S9B). From the spectra obtained (Figure 7C), there are clear differences between the Harmonic data and the data acquired using alternative surgical devices, especially in the *m*/*z* range of 600–1000 *m*/*z* where the complex mixture of triglyceride ions was observed, similarly to the negative ion data. There are also clear differences in the intensity of phospholipid ions observed in the *m*/*z* range of 700–800. In the *m*/*z* range of 600–700, high-abundant diglycerides were observed in all the spectra, something which was not observed in the negative mode data (*vide supra*). In the low *m*/*z* range of 100–550 (Supporting Information—Figure S10), a higher level of chemical noise was observed making the distinction and identification of ions more difficult. Despite the fact that the positive mode is not the most optimum mode when using the Harmonic device coupled to REIMS, especially in the lower mass range when higher chemical noise and interferences are observed (Figure S10), however it can be used for tissue classification showing a high degree of diagnostic accuracy (100%) (Figure S11). In addition, using the Harmonic device in the positive mode can be beneficial in ways where other specific groups of metabolites such as the triglycerides are observed. In this application of using the Harmonic device on biological tissue, the triglycerides observed in the positive mode have higher intensity compared to those observed in the negative mode; thus, when biological processes related to triglyceride metabolism are investigated, the positive mode could be favorable over the negative mode. A list of the most significant ions across the *m*/*z* range of 600–1000 can be found in the Supporting Information—Table Matrix 2.

### Application of the Harmonic Device Coupled to REIMS

These *ex vivo* experiments and validation work have demonstrated that the Harmonic device coupled to REIMS has the potential to be adapted into the surgical environment, where it can provide clinically important information used for tissue identification. The need for real-time chemical histology while using the Harmonic device is significant, and this energy device is the tool of choice for many surgeons undertaking abdominal cancer surgeries. Furthermore, given that it is not limited by organ system or tissue pathology (once the relevant specific metabolic profiles have been defined), there are a broad range of potential clinical applications. This would benefit any surgical procedure which requires margin detection, tissue phenotyping for therapy stratification, or anatomical identification to improve the safety and quality of the procedure (*e.g.,* tissue perfusion). Examples include the diagnosis of indeterminate peritoneal lesions in the context of advanced colorectal and ovarian cancer in order to plan resection strategies or “chemical staging” to predict markers of poor prognosis including the extent of lymph node metastasis which may require extended lymphadenectomy.^29^ Furthermore, it could be used as a chemical anatomic map to delineate planes such as ensuring complete mesorectal excision for rectal cancer (where the quality of specimen is directly correlated to oncological outcome).^30^ Finally, it provides a safety mechanism by alerting the surgeon to inadvertent injury to critical anatomical structures. Validation of REIMS coupled to the Harmonic device allows this technology to be applied laparoscopically without interfering with the surgical workflow or asking the surgeon to change to an unfavored device, giving them additional data on tissue which cannot be palpated or visualized with the naked eye. As robotic surgery increases in prevalence, this system can also be applied in this domain.^31^ This will promote high-quality minimally invasive surgery with the consequent improvements to patient safety and outcome. Finally, the high-intensity diglycerides and triglycerides serve as novel biomarker candidates for a range of benign and malignant conditions and provide mechanistic insights into disease etiology.

### Impact of Energy and Effect of Temperature on Cells and Tissue

The spectral differences seen between the monopolar electrosurgery and the Harmonic device may be explained by the impact of the electromagnetic energy on intracellular components and subsequently the effect of the temperature on cells and tissues. In the case of the monopolar electrosurgery where radiofrequency alternating current is deployed through a blade-shaped electrode, the thermal ablation is concentrated to the proximity of the point of contact due to current density effects. In course of thermal ablation, the power rapidly elevates the local intracellular temperature to approximately 700–800 °C causing massive expansion of the intracellular volume, as a result, the cell explodes creating a local plume of aerosol which is captured and analyzed by REIMS. In contrast, the Harmonic device operates by converting electrical energy into mechanical vibrations using a piezoelectric transducer.^32^ This mechanical energy advances to the instrument’s blade, generating heating through the dissipation of high-frequency longitudinal vibrations at 55,500 Hz. The elevated temperature changes the mechanical properties of tissues due to the thermal denaturation of structural proteins, which is followed by the mechanical fragmentation of more brittle tissue. While in the case of diathermy, heat is the main disruptive force, in the case of the harmonic, it is the mixture of heat and mechanical vibration. Consequently, the vibration will mostly aerosolize interstitial fluid without the disruption of biological membranes. The Harmonic scalpel generates temperatures in the range of 60–100 °C and thus avoids the charring and extensive chemical degradation of tissue components that can occur with thermal ablation at higher temperatures (>200 °C).^33^ Because this instrument operates in a lower temperature range than electrosurgery,^34^ water in the tissue (primarily interstitial fluid) will be heated by mechanical vibration and heat ablation, evaporate, and create an aerosol containing some extracellular components, but phospholipid bilayers will remain intact and will not disintegrate.^35^ Consequently, the number of phospholipids transferred to the instrument, ionized, and detected will be minimal, thus the spectral differences documented here, compared to other instruments of electrosurgical dissection. These experiments represent a preliminary feasibility study using the Harmonic device on porcine tissues and there is a biological plausibility that the spectra we have seen are representative of the lipid bilayers using the diathermy and interstitial fluid components using the Harmonic device. Further studies are required to microscopically examine this speculative finding to better understand the droplet formation mechanisms of the generated gaseous particles using the Harmonic device. It is interesting to note however that even though the number of phospholipids detected is lower when using the harmonic, we are still able to distinguish between the tissue types with a high accuracy (Figure S6).

## Conclusions

The Harmonic surgical scalpel is a next-generation energy device used for dissection during minimally invasive surgery for colorectal cancer. When coupled to REIMS, the Harmonic scalpel generates a unique spectral profile compared to monopolar diathermy and a CO_2_ laser, which can be accounted for with differences in the mechanism of aerosolization and droplet formation. It is able to differentiate the metabolic profiles of abdominal organs in a porcine model with a high accuracy largely based upon fatty acid and glycolipid ion abundances. When applied *in vivo*, this platform has the potential to give biological feedback to an operating surgeon, by defining tissue subtypes in real-time and allowing personalized clinical decisions to be made. This compelling example of precision surgery is a platform that can be applied to minimally invasive and open procedures for both oncologic and nononcologic surgery.

## Supporting Information Available

Surgical tools and generator settings used for the pilot *ex vivo* study; modified Harmonic scalpel; PCA plots and associated spectra of different surgical tools on porcine tissues; loading plots of the first two components in the *m*/*z* range of 100–1000 in the negative mode; box plots of different surgical instruments in the *m*/*z* range of 100–550; pork liver REIMS spectra in the negative mode in the *m*/*z* range of 600–800 and 800–1000; box plots of different surgical instruments in the *m*/*z* range of 600–1000; examples of MS/MS spectra of *m*/*z* 885.55 and *m*/*z* 701.50 in the negative mode; examples of MS/MS spectra of [PG (36:2)-H]; loading plots of the first two components in the *m*/*z* range of 100–1000 in the positive mode; pork liver REIMS spectra in the positive mode in the *m*/*z* range of 100–1000; confusion matrix of the Harmonic device in the positive and negative mode on pork liver tissue;tentative identifications and MSMS data in the *m*/*z* range of 100–1000 in the negative mode; and tentative identifications and MSMS data in the *m*/*z* range of 600–1000 in the positive mode (PDF)
