# Supplementary material for: Validation of Ultrasonic Harmonic Scalpel for Real-Time Tissue Identification Using Rapid Evaporative Ionization Mass Spectrometry
Source: Anal Chem. 2021 Mar 31;93(14):5906–16. doi: 10.1021/acs.analchem.1c00270 (PMC8153397; doi:10.1021/acs.analchem.1c00270)
Supplement: Supplementary file 1 — ac1c00270_si_001.pdf [file ac1c00270_si_001.pdf]

Supporting information

Validation of ultrasonic Harmonic® scalpel for real-time tissue identification using Rapid Evaporative Ionisation Mass Spectrometry (REIMS)

Eftychios Manoli[a], Sam Mason[a], Lauren Ford[a], Afeez Adebesin[a], Zsolt Bodai[b], Ara Darzi[a], James Kinross[a], Zoltan Takats*[b]

[a]
Department of Surgery and Cancer,
Imperial College London
St Marys Hospital, London, W2 1NY, United Kingdom

[b]
Department of Metabolism, Digestion and Reproduction,
Imperial College London
South Kensington Campus, London, SW7 2AZ, United Kingdom

*Corresponding Author: Prof. Zoltan Takats, Email z.takats@imperial.ac.uk

Table of Contents

Table S1: Surgical tools and generator settings used for the pilot ex vivo study **2**

Figure S1: Modified harmonic scalpel **2**

Figure S2: PCA plots and associated spectra of different surgical tools on porcine tissues **5**

Figure S3: Loading plots of the first two components om the *m/z* range of 100-1000 in negative mode........ **6**

Figure S4: Box plots of different surgical instruments in the *m/z* range of 100-550 .... **7**

Figure S5: Pork liver REIMS spectra in negative mode in the *m/z* range of 600-800 and 800-1000........ **8**

Figure S6: Box plots of different surgical instruments in the *m/z* range of 600-1000........ **9**

Figure S7: Examples of MS/MS spectra of *m/z* 885.55 and *m/z* 701.50 in negative mode **10**

Figure S8: Examples of MS/MS spectra of PG (36:2)-H]$^-$ **12**

Figure S9: Loading plots of the first two components in the *m/z* range of 100-1000 in positive mode **13**

Figure S10: Pork liver REIMS spectra in positive mode in the *m/z* range of 100-1000 .... **14**

Figure S11: Confusion matrix of Harmonic device in positive and negative mode on pork liver tissue **14**

Table Matrix 1: Tentative identifications and MSMS data in the *m/z* range of 100-1000 in negative mode **15**

Table Matrix 2: Tentative identifications and MSMS data in the *m/z* range of 600-1000 in positive mode........ **17**

Table S1: Surgical tools and generator settings used for the pilot ex vivo study

Surgical Instrument	Mode	Power (W)	Handpiece/Electrode parameters	Mechanism
Monopolar Electrosurgery	Cut	20	Short straight standard blade (1cm)	Joule heating
Monopolar Electrosurgery	Coagulative	20	Short straight standard blade (1cm)	Joule heating
CO_2 Laser	Superpulse	3	Flexible selected fibre delivery system, 30psi, He gas used	Radiative heating
Harmonic Scalpel	Cut	5	Tapered Tip Blade	Vibrational energy dissipation

Figure S1: Modified harmonic scalpel

A 3 mm i.d (PTFE) tubing was attached at the end of the shaft of the harmonic instrument, to allow efficient aspiration of the surgical aerosol into the mass spectrometer. The tube was secured on the shaft using a Transpore Surgical Tape (2.5cm x 9.14m).

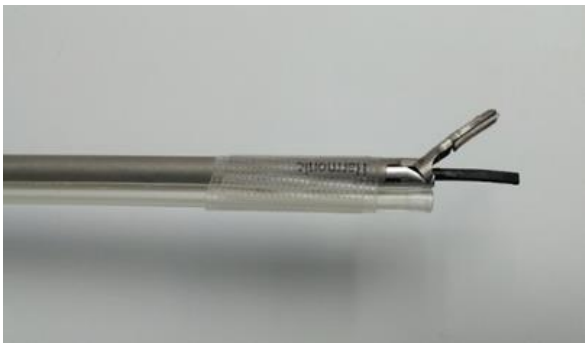

Figure S2: PCA plots and associated spectra of different surgical tools on porcine tissues

PCA plots of different surgical tools on porcine muscle A) colon B) and small intestine C) in the *m/z* range of 100-550 and 600-1000 showing a good separation between the harmonic scalpel and the other instruments. Each point represents a sample burn. The tolerance ellipse of the two-dimensional score plot (PC1 and PC2) was based on Hotelling's T2 with significant level set to 0.05. Comparison of averaged MS spectra acquired on muscle, colon and small intestine tissue in negative mode using a Xevo S2-XS instrument (Waters Corporation, UK). The magnified lipid in the *m/z* range of 600-1000 shows significant spectra differences for each tool on each tissue type.

A) Muscle

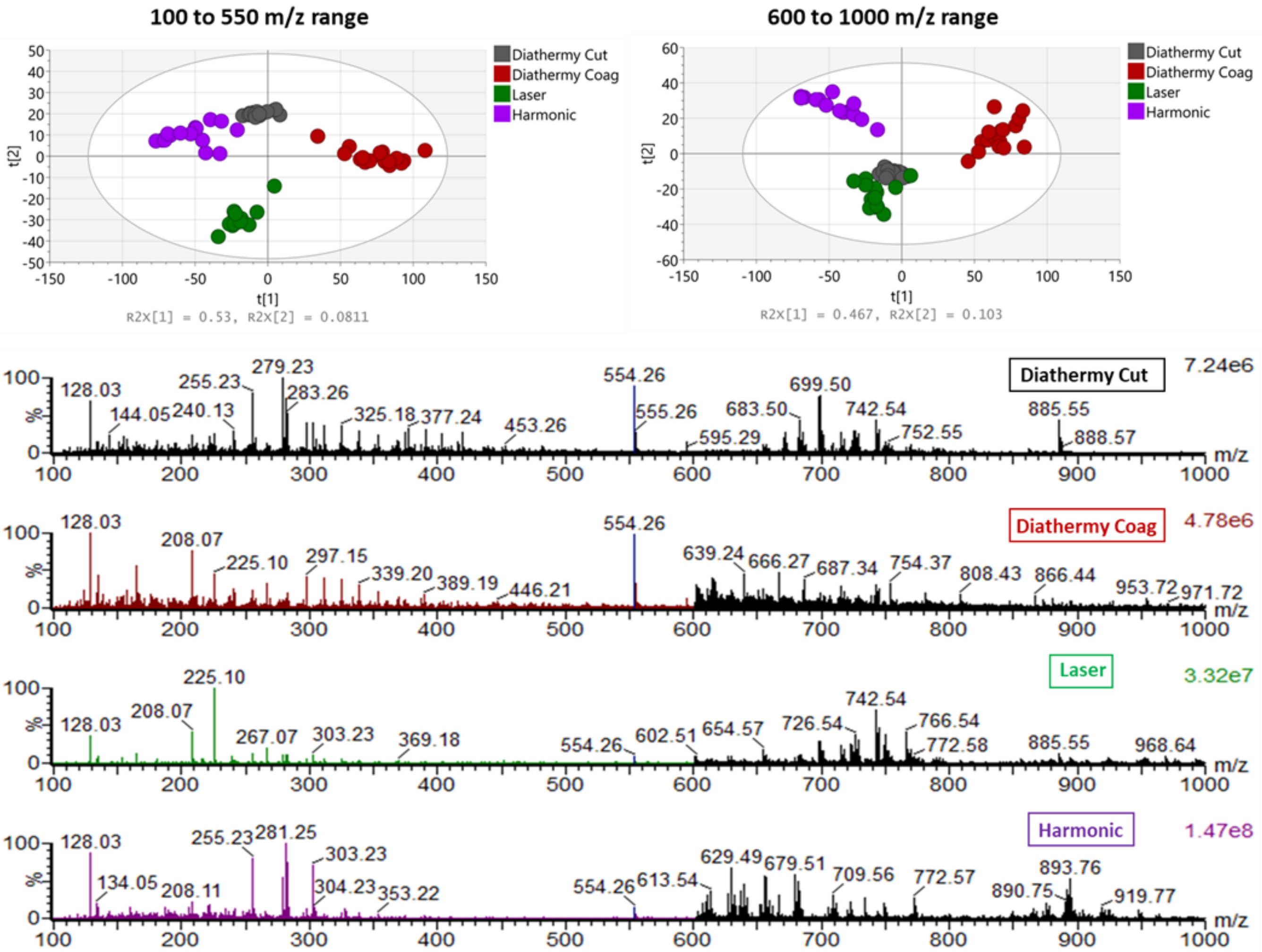

B) Colon

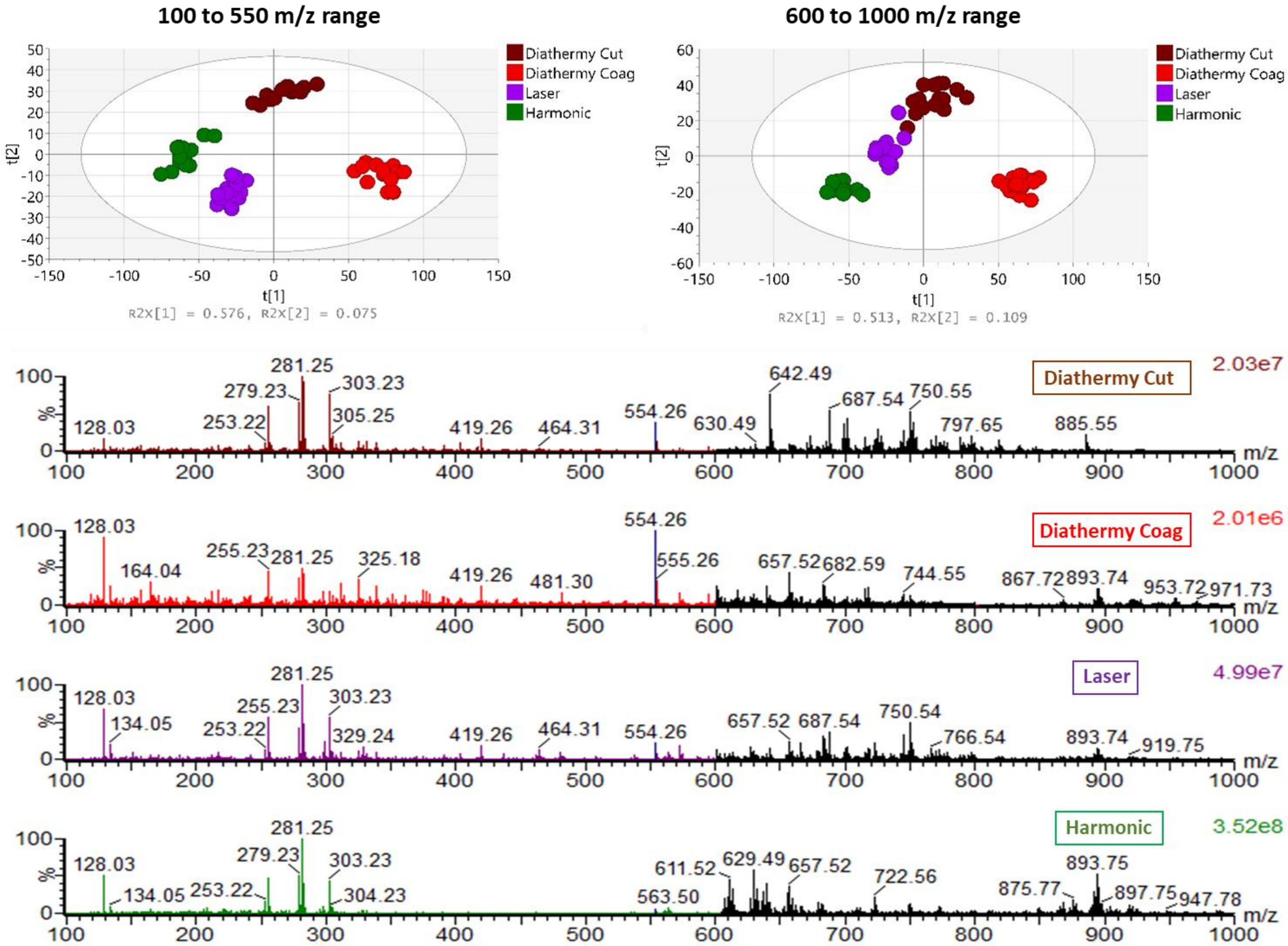

100 to 550 m/z range
600 to 1000 m/z range
Diathermy Cut
Diathermy Coag
Laser
Harmonic
t[1]
t[2]
R2X[1] = 0.576, R2X[2] = 0.075
R2X[1] = 0.513, R2X[2] = 0.109
Diathermy Cut
2.03e7
128.03
253.22
279.23
281.25
303.23
305.25
419.26
464.31
554.26
630.49
642.49
687.54
750.55
797.65
885.55
Diathermy Coag
2.01e6
128.03
164.04
255.23
281.25
325.18
419.26
481.30
554.26
555.26
657.52
682.59
744.55
867.72
893.74
953.72
971.73
Laser
4.99e7
128.03
134.05
253.22
255.23
281.25
303.23
329.24
419.26
464.31
554.26
657.52
687.54
750.54
766.54
893.74
919.75
Harmonic
3.52e8
128.03
134.05
253.22
279.23
281.25
303.23
304.23
563.50
611.52
629.49
657.52
722.56
875.77
893.75
897.75
947.78
m/z
%

C) Small Intestine

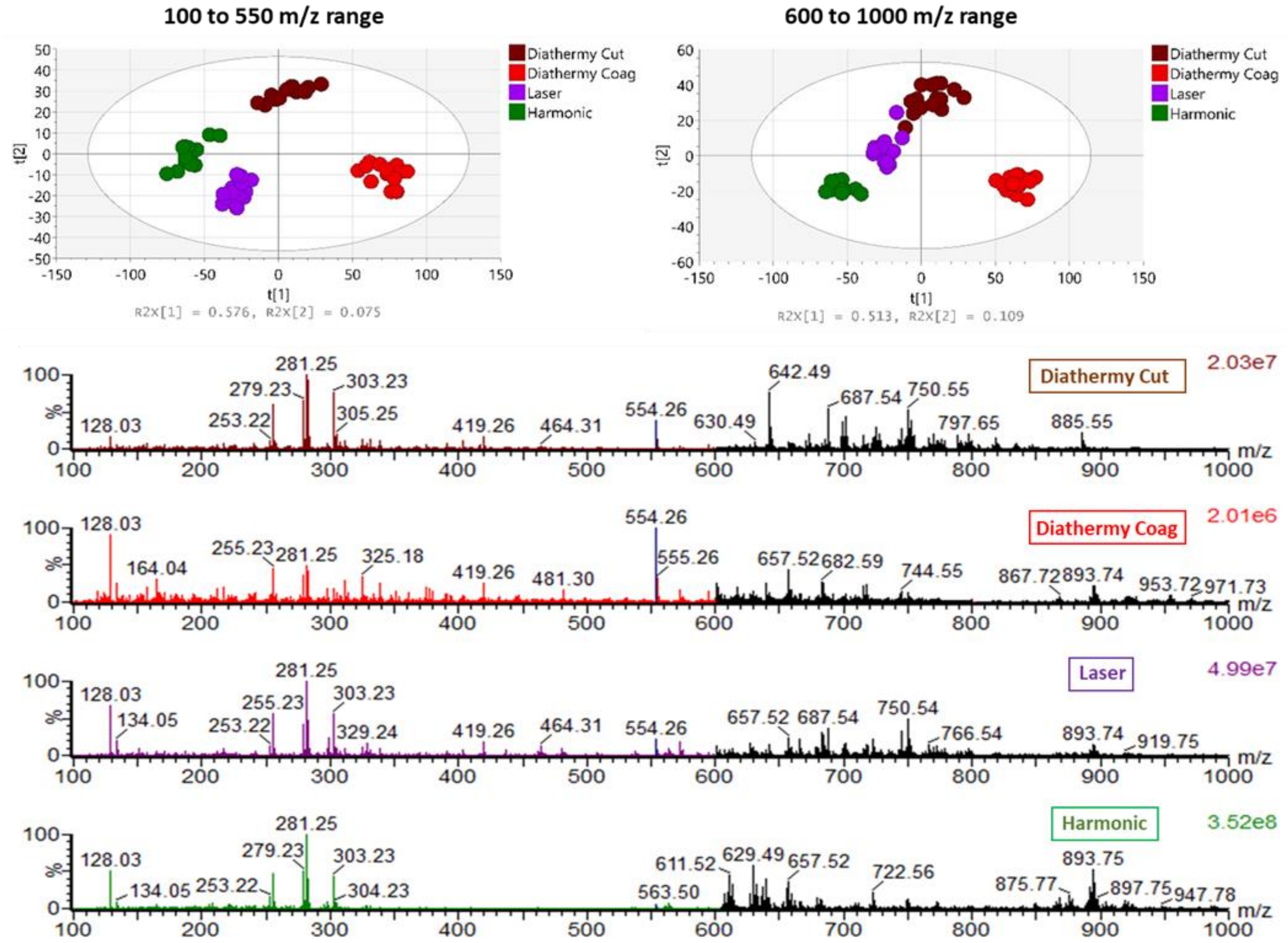

Figure S3: Loading plots of the first two components om the *m/z* range of 100-1000 in negative mode

Loading plots of the first two components in the range of 100-550 A) with PC1=63.2% and PC2=16.94% and 600-1000 B) with PC1=62.01% and PC2=16.6%. The plots were made on AMX software (Waters, Hungary). Negative ionisation mode was used. Different peaks across the *m/z* mass range 100-1000, contribute to the separation of each group including different fatty acids, glycerolipids and glycerophospholipids

A) 100-550 *m/z* range

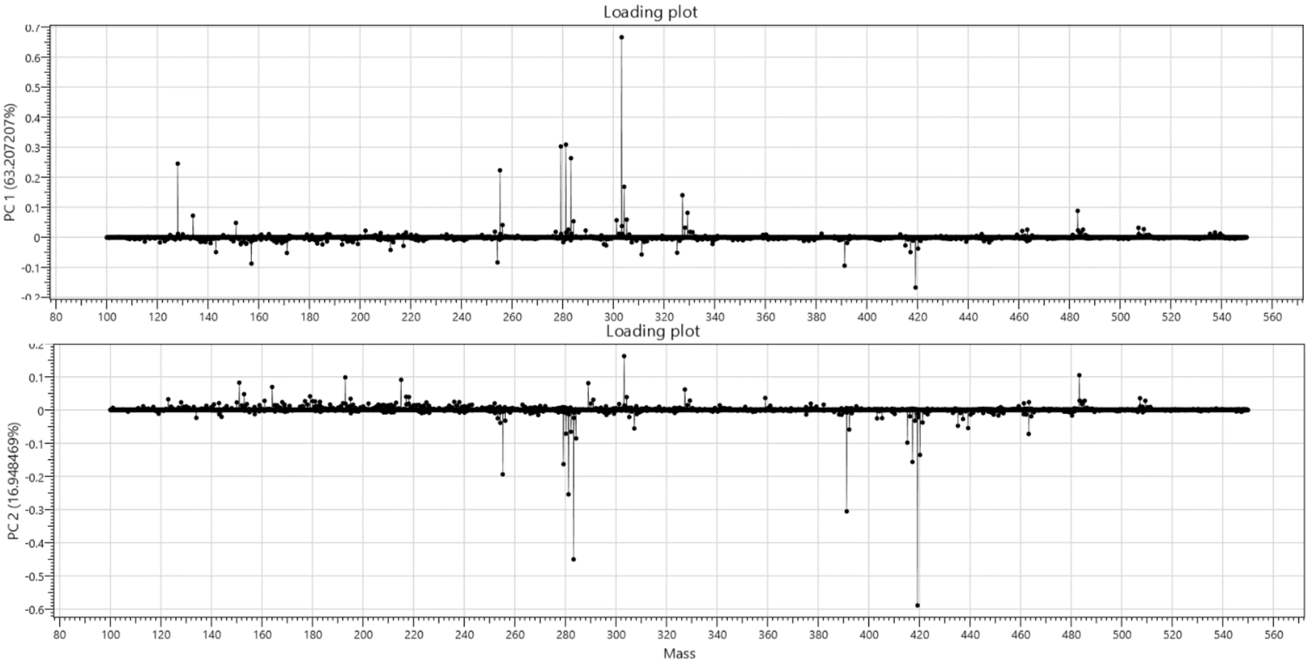

B) 600-1000 *m/z* range

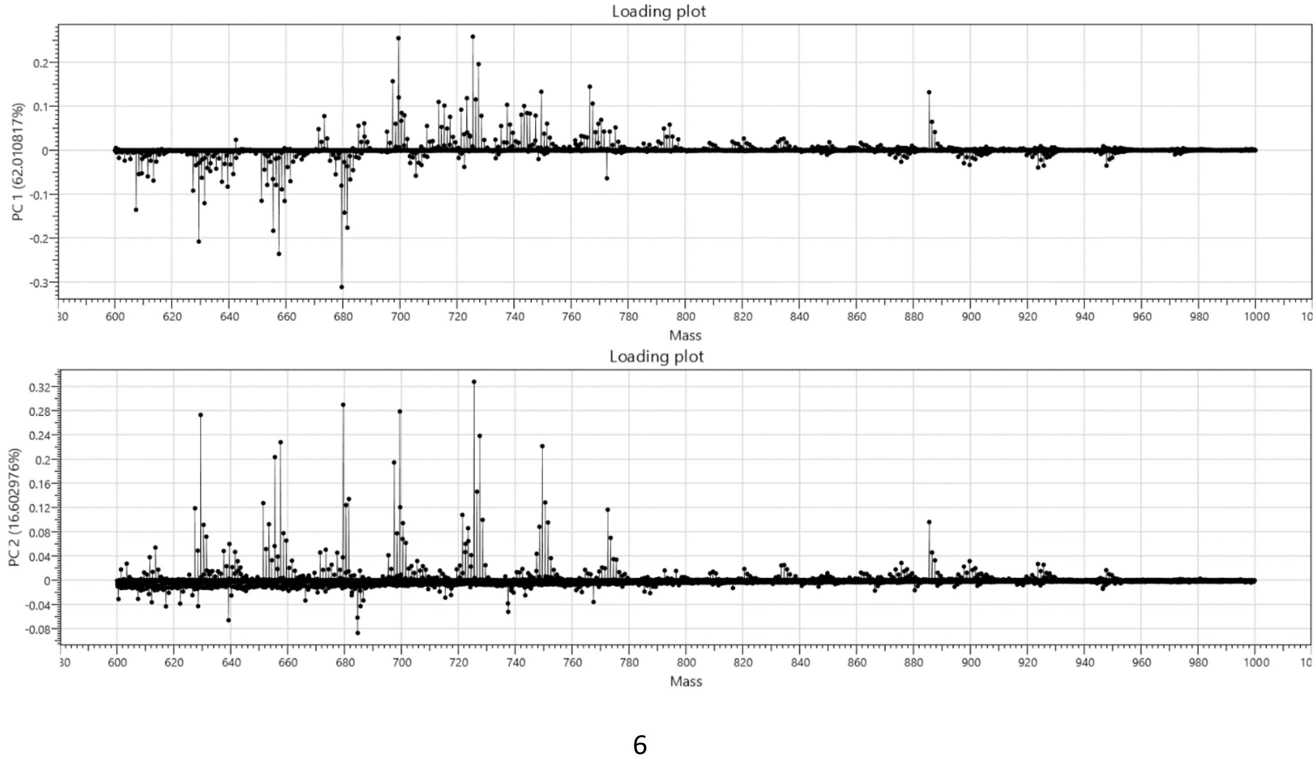

Figure S4: Box plots of different surgical instruments in the *m/z* range of 100-550

Comparison of selected peaks that are significantly different between the diathermy cut/coag, laser and harmonic surgical tools in the *m/z* range of 100-550 using box plots (FDR corrected p <0.05). Anova was made in R Studio and every surgical tool's p value was calculate against the harmonic data as a reference point. The groups cut/coag refer to diathermy data in cutting and coagulation mode

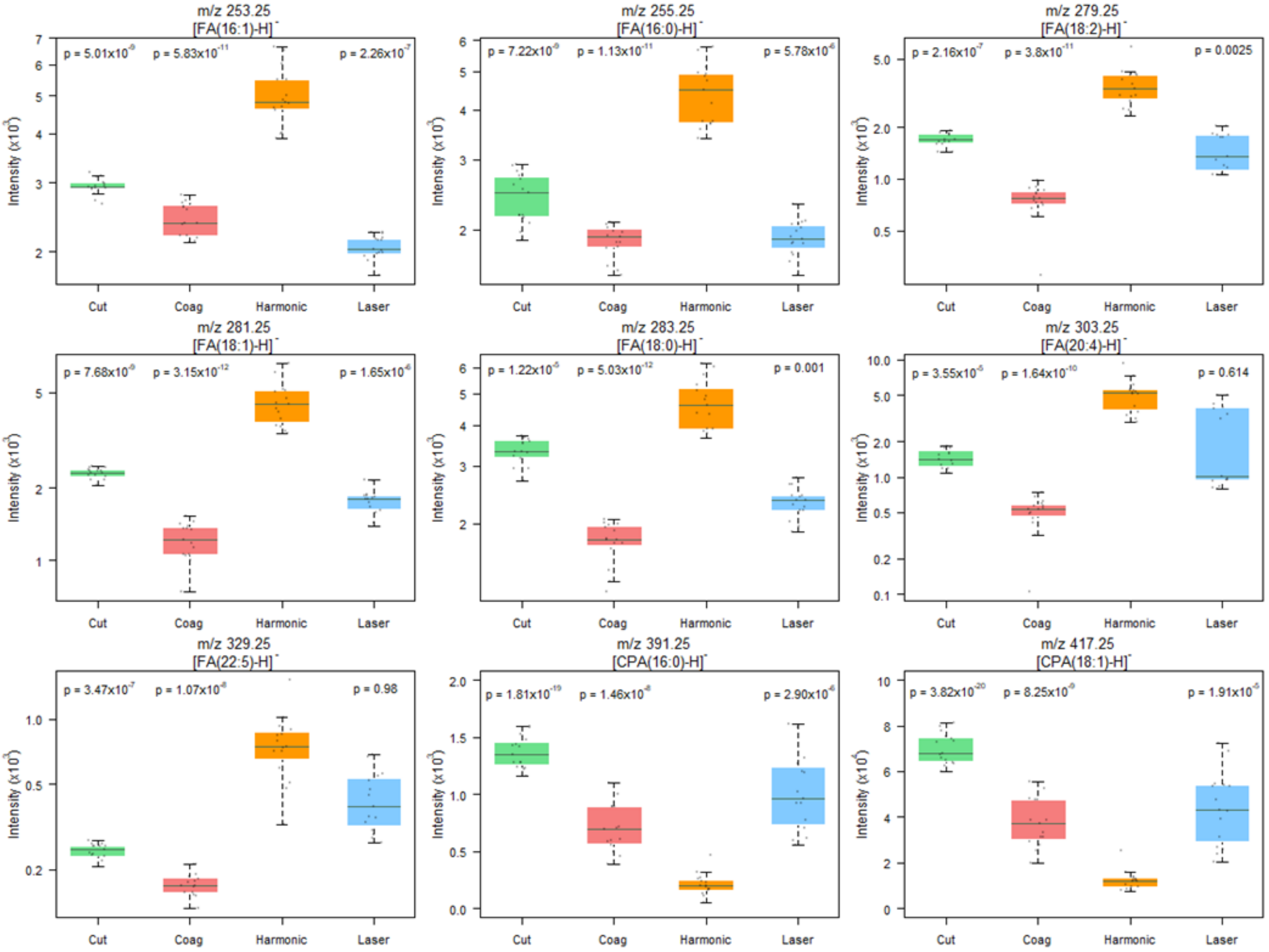

Figure S5: Pork liver REIMS spectra in negative mode in the *m/z* range of 600-800 and 800-1000

A) Expanded MS spectra acquired on pork liver using different surgical instrument in the *m/z* range of 600-800. The first three instruments show great similarities in the ions observed in the lipid range whereas the harmonic's spectra show completely different spectral patterns, where high abundant diglycerides are predominantly observed. B) Expanded MS spectra acquired on pork liver using different surgical instrument in the *m/z* range of 800-1000. Same distinctive phospholipid ions (such as Phosphatidyl-Inositol (20:4_18:0)-H]- at *m/z* 885.55) were observed in the first three instruments, compared to the harmonic spectra where high intensity triglycerides are seen

A) 600-800 *m/z* range

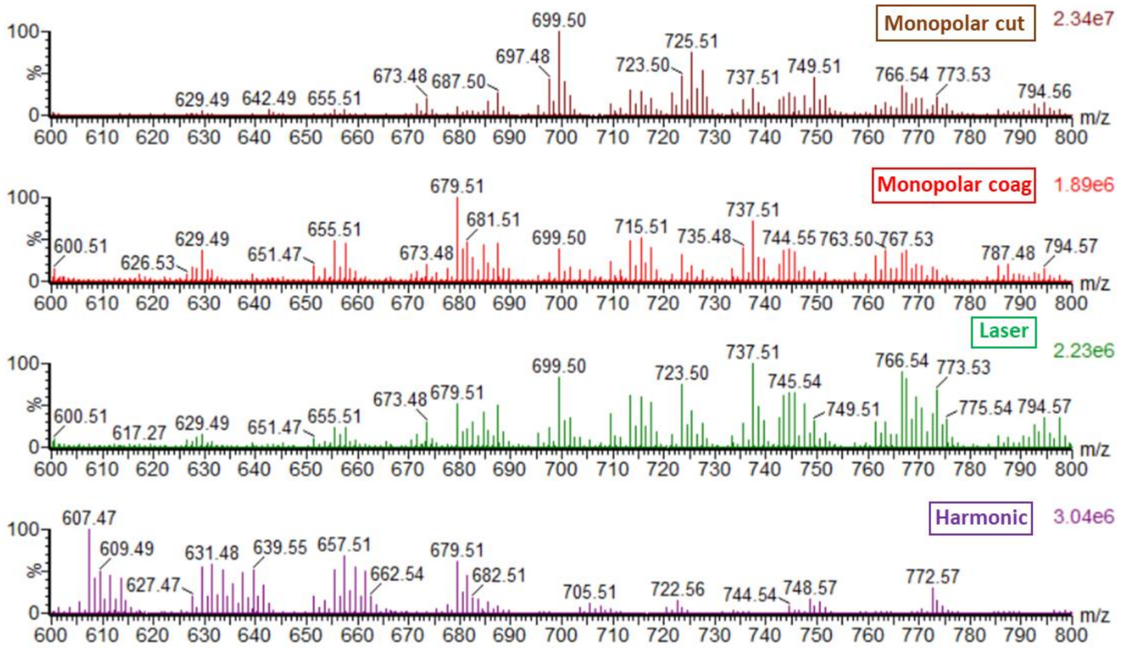

B) 800-1000 *m/z* range

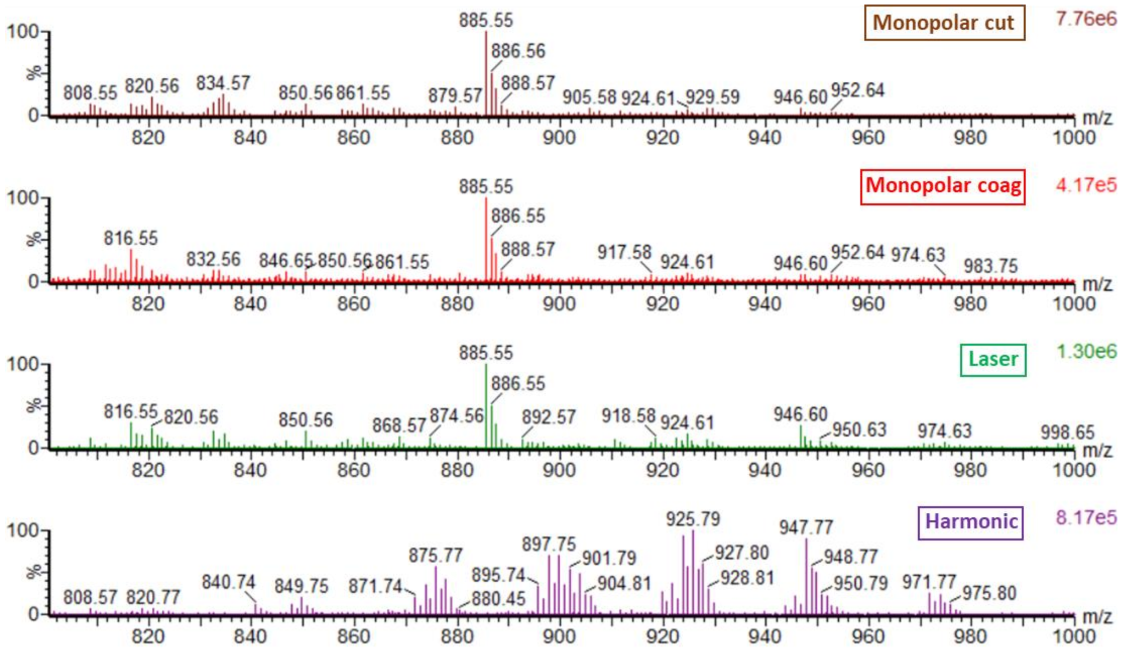

Figure S6: Box plots of different surgical instruments in the *m/z* range of 600-1000

Comparison of selected peaks that are significantly different between the diathermy cut/coag, laser and harmonic surgical tools in the *m/z* range of 600-1000 using box plots (FDR corrected $p < 0.05$). Anova was made in R Studio and every surgical tool's p value was calculate against the harmonic data as a reference point. The groups cut/coag refer to diathermy data in cutting and coagulation mode. In some cases, p values were plotted at the bottom of the box plots for better visualisation

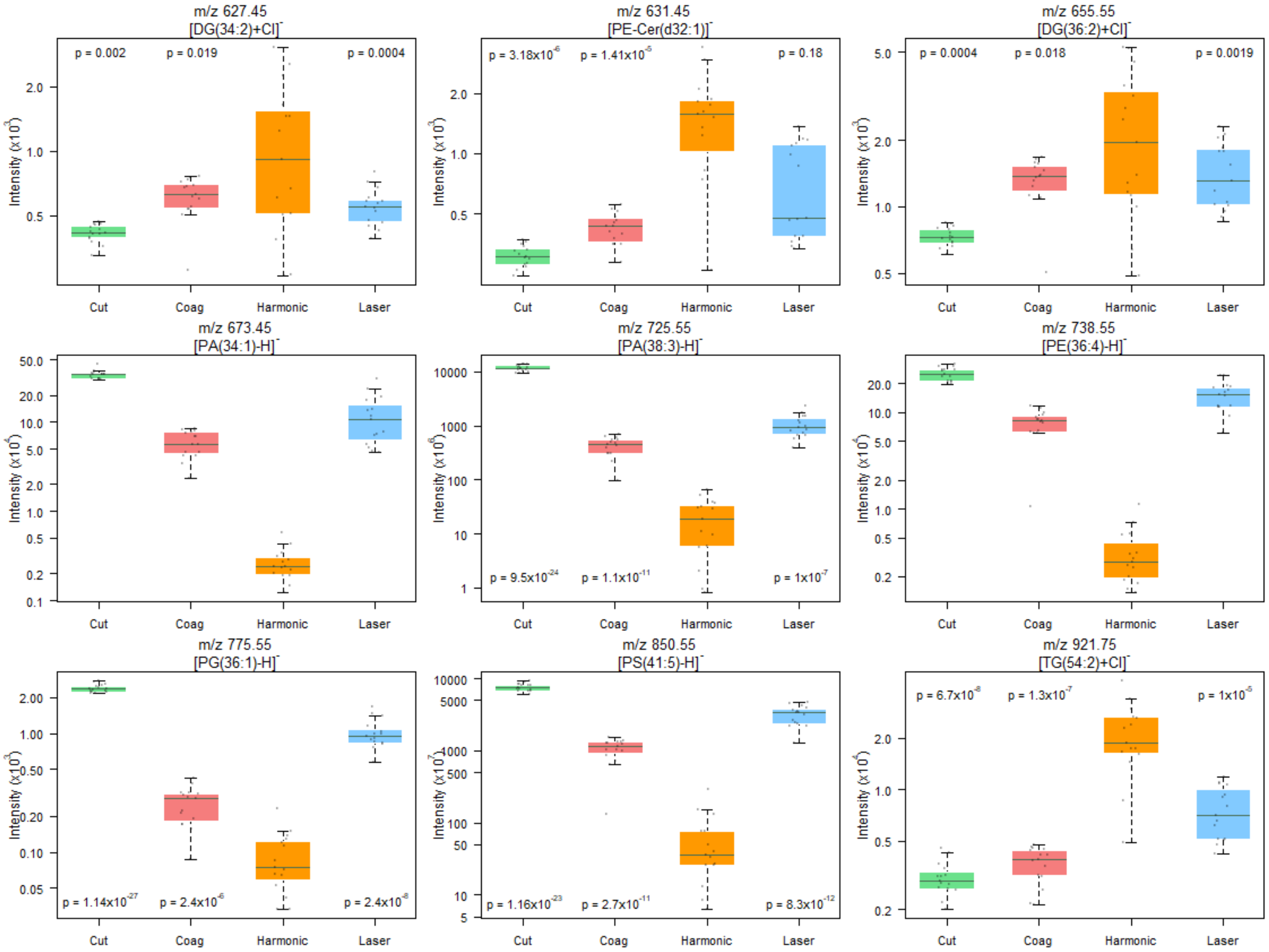

Figure S7: Examples of MS/MS spectra of *m/z* 885.55 and *m/z* 701.50 in negative mode

Comparison MS/MS spectra of A) *m/z* 885.55 and B) *m/z* 701.50 of the pork liver extract in negative mode. Collison energies were used at 40V and 30V respectively (*PA - Phosphatidic acid, PI - Phosphatidylinositol)*

A) PA (18:1-18:0)-H]$^-$

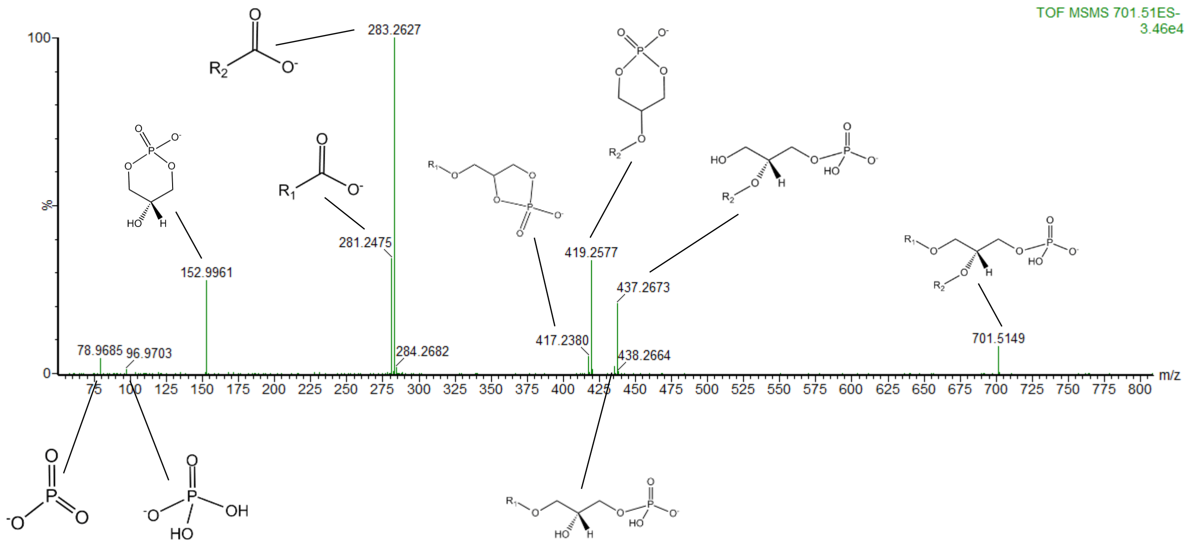

B) PI (20:4-18:0)-H]$^-$

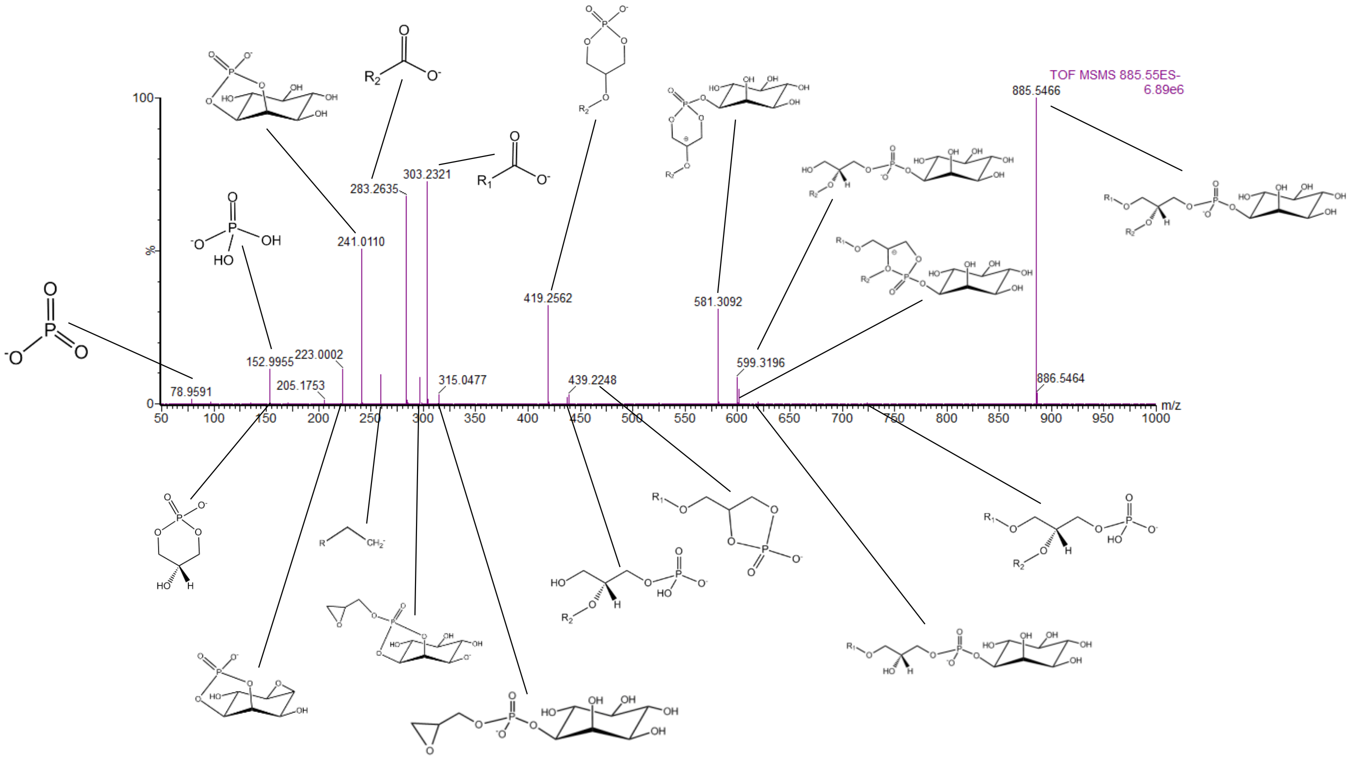

Figure S8: Examples of MS/MS spectra of PG (36:2)-H]⁻ data where more than one possible lipid structure

A) (18:1-18:1)-H]⁻ with CE (ramp 30-50V) (*PG – Phosphatidylglycerol*)

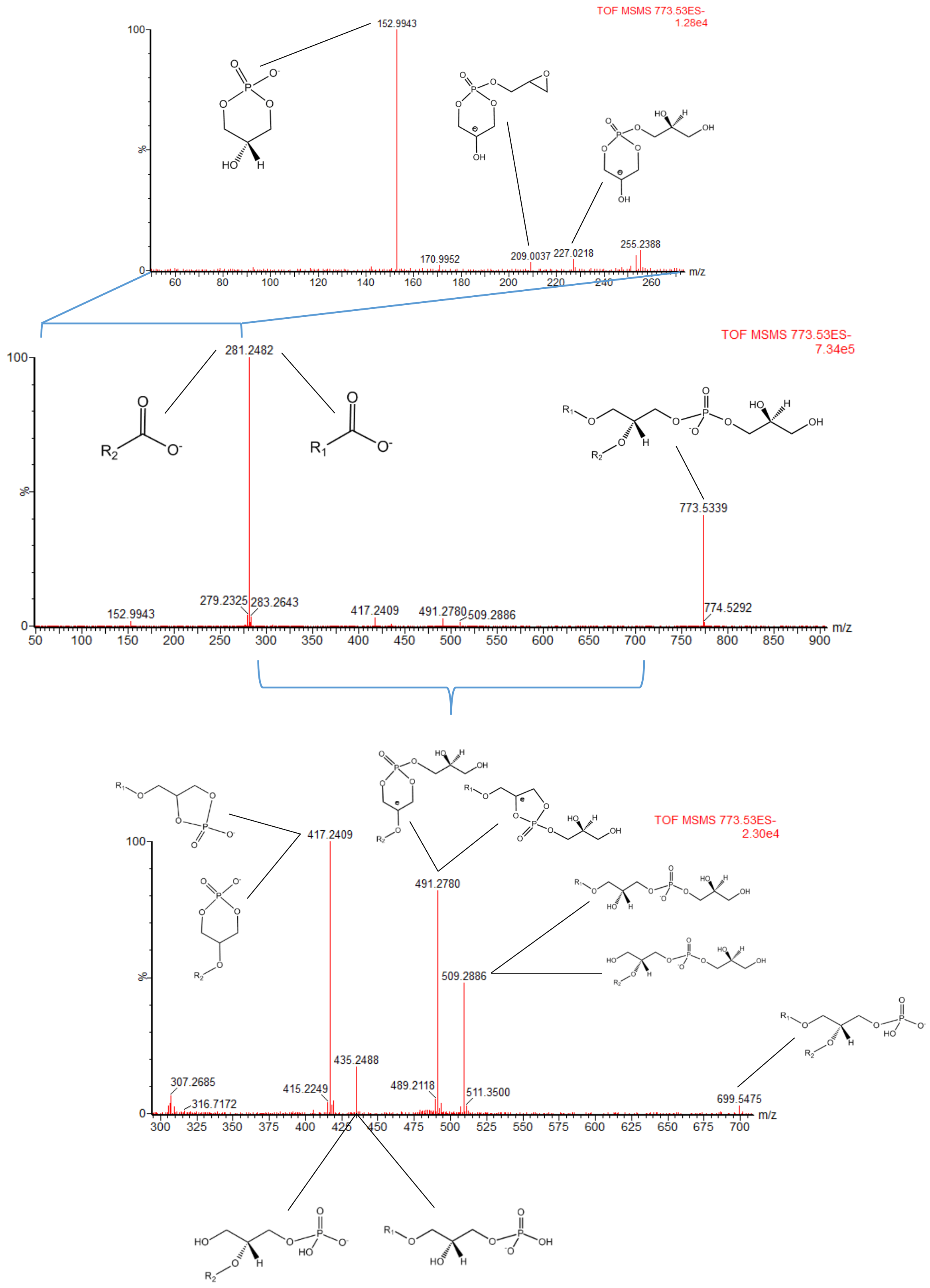

B) PG (18:2-18:0)-H]- with CE (ramp 30-50V)

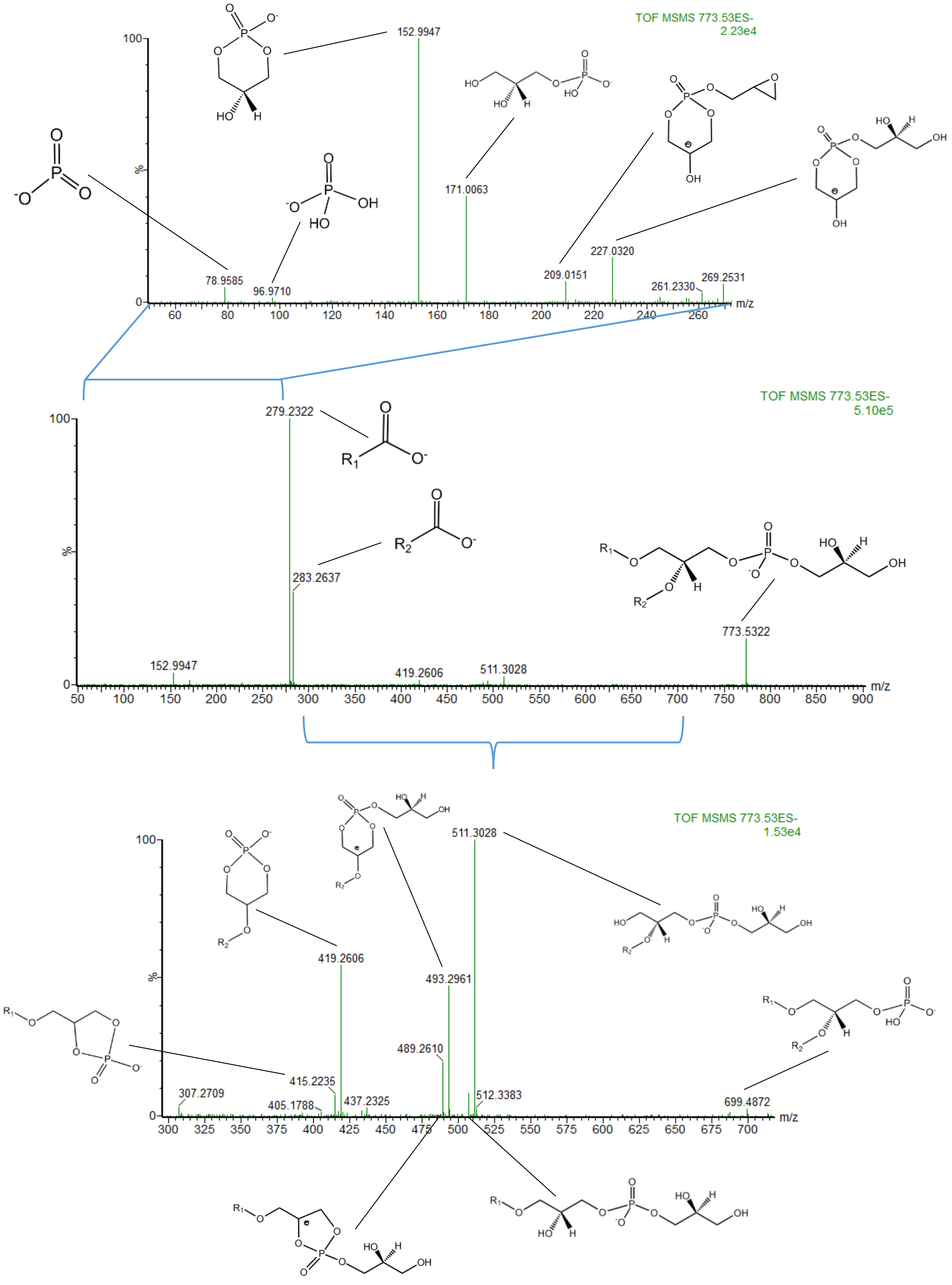

Figure S9: Loading plots of the first two components in the *m/z* range of 100-1000 in positive mode

A) *m/z* range of 100-550

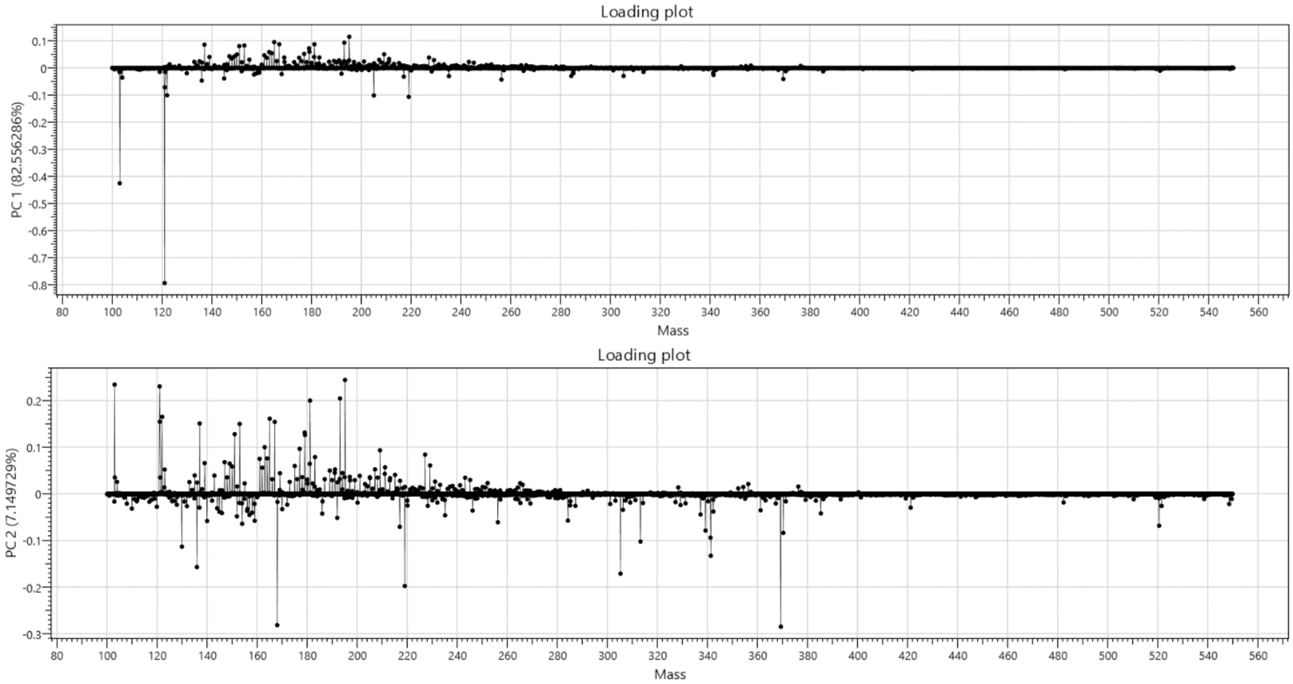

B) *m/z* range of 600-1000

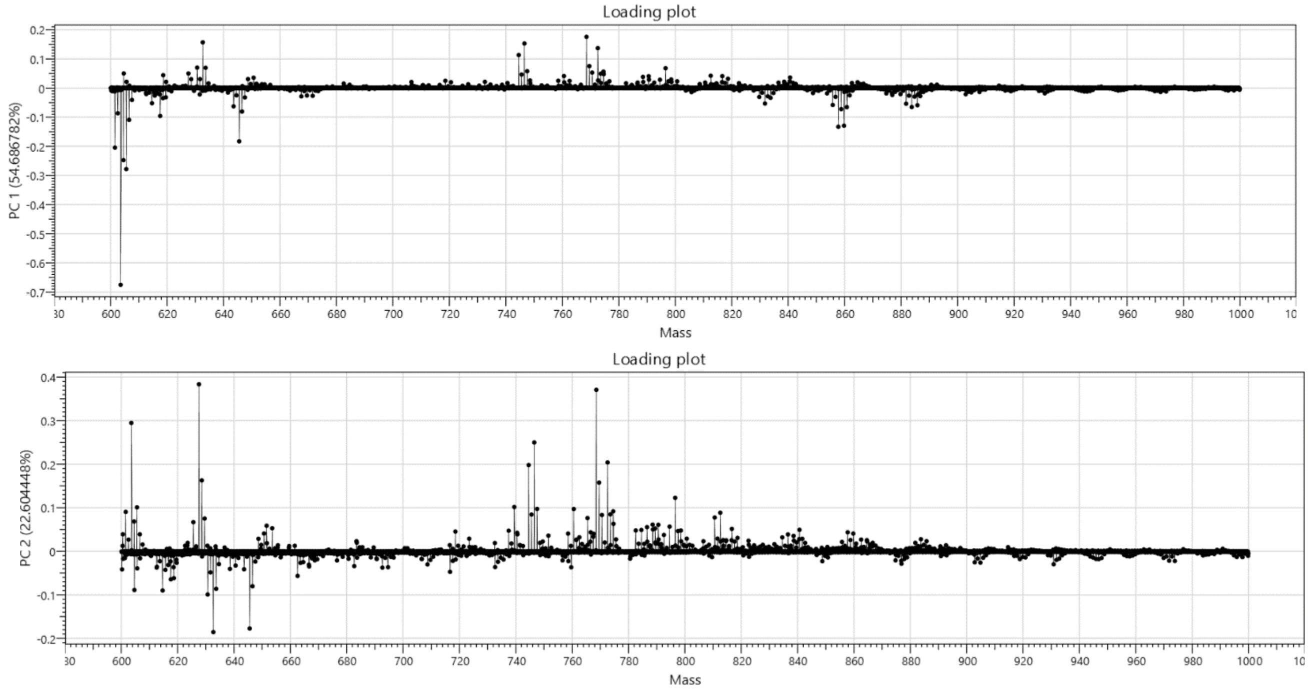

Figure S10: Pork liver REIMS spectra in positive mode in the *m/z* range of 100-1000

REIMS spectra acquired on pork liver positive mode using different surgical instruments in the *m/z* range of 100-1000. For better visualisation the *m/z* range of 600-1000 was magnified.

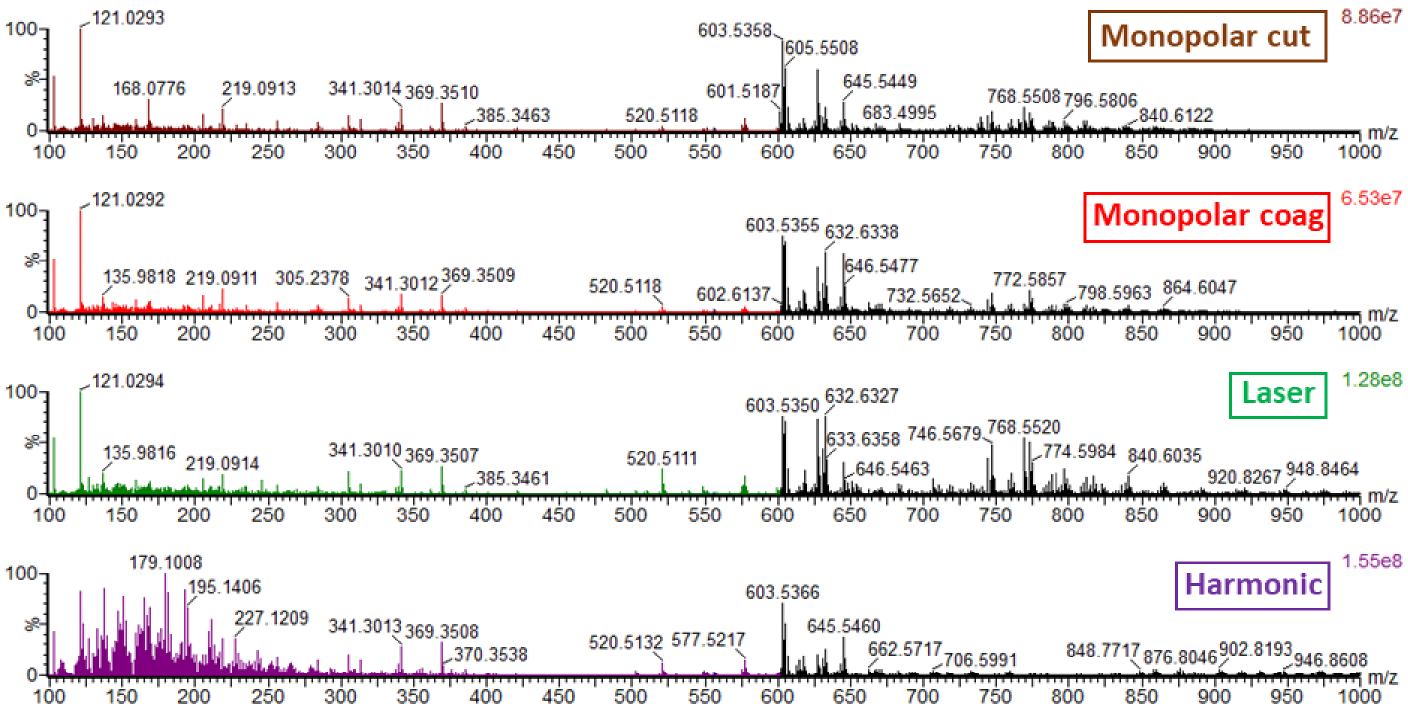

Figure S11: Confusion matrix of Harmonic device in positive and negative mode on pork liver tissue

Comparison of tissue classification using the harmonic device in positive and negative ion mode on pork liver in the *m/z* range of 600-1000. 100% overall diagnostic accuracy was observed using leaving on spectrum out cross validation.

	1	2	3	4	5	6
1	M2	**Members**	**Correct**	**Harmonic Pos**	**Harmonic Neg**	**No class (YPred <= 0)**
2	**Harmonic Pos**	20	100%	20	0	0
3	**Harmonic Neg**	20	100%	0	20	0
4	**No class**	0		0	0	0
5	Total	40	100%	20	20	0
6	Fisher's prob.	7.3e-12				

Table Matrix 1: Tentative identifications and MSMS data in the *m/z* range of 100-1000 in negative mode

Tentative identifications and MSMS data of peaks in the *m/z* range of 100 to 1000 in negative mode. Accurate masses of candidate *m/z* peaks, to four decimal places after lock mass correction (Leucine Enkephalin at *m/z* 554.2615) were used to interrogate the LIPID MAPS database (www.lipidmaps.org) with the highest ranked match, based on delta value (< 0.05). A list is given below whether the *m/z* peak observed is more abundant in the Harmonic or diathermy REIMS data. (FA- Fatty acid, *Cer-Ceramide, DG-Diglyceride, PE-Phosphatidylethanolamine, PI-Phosphatidylinositol, TG-Triglyceride, PG – Phosphatidylglycerol, PA - Phosphatidic acid, PS-Phosphatidylserine, DGCC- Diglyceride Carboxyl Choline, HexCer-Hexosyl Ceramide)*

	m/z observed	ID	Adduct	Delta value	ppm error	m/z theoretical	Higher abundance	MSMS	Fatty acid composition	Collison energy (V)
1	253.2164	FA(16:1)□	[M-H]-	-0.0009	-3.55	253.2173	Harmonic	n/a	n/a	n/a
2	254.2484	Sph(m16:1)	[M-H]-	-0.0005	-1.97	254.2489	Diathermy	n/a	n/a	n/a
3	255.2332	FA(16:0)	[M-H]-	0.0002	0.78	255.233	Harmonic	n/a	n/a	n/a
4	279.2328	FA(18:2)	[M-H]-	-0.0002	-0.72	279.233	Harmonic	n/a	n/a	n/a
5	281.2489	FA(18:1)	[M-H]-	0.0003	1.07	281.2486	Harmonic	n/a	n/a	n/a
6	283.2635	FA(18:0)	[M-H]-	-0.0008	-2.82	283.2643	Harmonic	n/a	n/a	n/a
7	301.2162	FA(20:5)	[M-H]-	-0.0011	-3.65	301.2173	Harmonic	n/a	n/a	n/a
8	303.2334	FA(20:4)	[M-H]-	0.0004	1.32	303.233	Harmonic	n/a	n/a	n/a
9	305.251	FA(20:3)	[M-H]-	0.0024	7.86	305.2486	Harmonic	n/a	n/a	n/a
10	307.2633	FA(20:2)	[M-H]-	-0.001	-3.25	307.2643	Diathermy	n/a	n/a	n/a
11	325.1836	FA(21:6(Ep,cyclo))	[M-H]-	0.0027	8.30	325.1809	Diathermy	n/a	n/a	n/a
12	327.2343	FA(22:6)	[M-H]-	0.0013	3.97	327.233	Harmonic	n/a	n/a	n/a
13	329.2456	FA(22:5)	[M-H]-	-0.003	-4.94	329.2486	Harmonic	n/a	n/a	n/a
14	607.4725	FA_Dimer(40:8)	[M-H]-	-0.0001	-0.16	607.4726	Harmonic	YES	n/a	n/a
15	613.5333	DG(O-33:0)	[M+Formate]-	-0.008	-13.04	613.5413	Harmonic	n/a	n/a	n/a
16	627.476	DG(34:2)	[M+Cl]-	-0.0001	-0.16	627.4761	Harmonic	YES	n/a	n/a
17	629.4897	DG(34:1)	[M+Cl]-	-0.002	-3.18	629.4917	Harmonic	n/a	n/a	n/a
18	631.4821	PE-Cer(d32:1)	[M-H]-	0	0.00	631.4821	Harmonic	n/a	n/a	n/a
19	633.4978	PE-Cer(d32:0)	[M-H]-	0.0001	0.16	633.4977	Harmonic	YES	n/a	n/a
20	637.539	DG(O-35:2)	[M+Formate]-	-0.0023	-3.61	637.5413	Harmonic	YES	n/a	n/a
21	639.5548	DG(O-35:1)	[M+Formate]-	-0.0021	-3.28	639.5569	Harmonic	n/a	n/a	n/a
22	642.5731	Cer(t37:0)	[M+Formate]-	0.0053	8.25	642.5678	Diathermy	n/a	n/a	n/a
23	643.4908	PE-Cer(d33:2)	[M-H]-	0.0088	13.68	643.482	Diathermy	YES	PE_Cer(d15:2_18:0)	n/a
24	645.4904	PA(O-33:1)	[M-H]-	0.0039	6.04	645.4865	Harmonic	n/a	n/a	n/a
25	651.4759	DG(36:4)	[M+Cl]-	-0.0002	-0.31	651.4761	Harmonic	YES	n/a	n/a
26	653.4831	DG(36:3)	[M+Cl]-	-0.0086	-13.16	653.4917	Harmonic	YES	n/a	n/a
27	655.506	DG(36:2)	[M+Cl]-	-0.0014	-2.14	655.5074	Harmonic	n/a	n/a	n/a
28	659.522	PE-Cer(d34:1)	[M-H]-	0.0086	13.04	659.5134	Harmonic	n/a	n/a	n/a
29	661.5386	PE-Cer(d34:0)	[M-H]-	0.0096	14.51	661.529	Harmonic	n/a	n/a	n/a
30	671.4655	PA(34:2)	[M-H]-	-0.0002	-0.30	671.4657	Diathermy	YES	PA(16:0_18:2)	30
31	673.481	PA(34:1)	[M-H]-	-0.0004	-0.59	673.4814	Diathermy	YES	PA(16:0_18:1)	30
32	674.4849	PE(31:1)	[M-H]-	0.0083	12.31	674.4766	Diathermy	YES	PE(16:1_16:0)	30
33	677.4883	DG(38:5)	[M+Cl]-	-0.0034	-5.02	677.4917	Harmonic	YES	n/a	n/a
34	679.5068	DG(38:4)	[M+Cl]-	-0.0006	-0.88	679.5074	Harmonic	n/a	n/a	n/a
35	685.4828	PA(35:2)	[M-H]-	0.0014	2.04	685.4814	Diathermy	n/a	n/a	n/a
36	687.4974	PA(35:1)	[M-H]-	0.0004	0.58	687.497	Diathermy	n/a	n/a	n/a
37	695.4651	PA(36:4)	[M-H]-	-0.0006	-0.86	695.4657	Diathermy	YES	PA(16:0_20:4)	30
38	697.4814	PA(36:3)	[M-H]-	0	0.00	697.4814	Diathermy	YES	PA(18:1_18:2)<br>PA(16:0_20:3)	30
39	699.4971	PA(36:2)<br>PE(34:1)	[M-H]-<br>[M-NH3]-	0.0001	0.14	699.497	Diathermy	n/a	n/a	n/a
40	701.5093	PA(36:1)	[M-H]-	-0.0034	-4.85	701.5127	Diathermy	YES	PA(18:1_18:0)	30
41	703.5073	DG(40:6)	[M+Cl]-	-0.0001	-0.14	703.5074	Harmonic	n/a	n/a	n/a
42	705.5184	DG(40:5)	[M+Cl]-	-0.0046	-6.52	705.523	Harmonic	n/a	n/a	n/a
43	709.4805	PA(37:4)	[M-H]-	-0.0009	-1.27	709.4814	Diathermy	n/a	n/a	n/a
44	709.5514	DG(40:3)	[M+Cl]-	-0.0029	-4.09	709.5543	Harmonic	n/a	n/a	n/a
45	713.5121	PA(37:2)	[M-H]-	-0.0006	-0.84	713.5127	Diathermy	n/a	n/a	n/a
46	716.5213	PE(34:1)	[M-H]-	-0.0023	-3.21	716.5236	Diathermy	YES	PE(16:0_18:1)<br>PE(16:1_18:0)	30
47	717.5092	PG(O-33:2)	[M-H]-	0.0016	2.23	717.5076	Diathermy	YES	n/a	n/a
48	722.5575	HexCer(d36:3)	[M-H]-	-0.0001	-0.14	722.5576	Harmonic	n/a	n/a	n/a
49	723.4965	PA(38:4)	[M-H]-	0	0.00	723.4965	Diathermy	YES	PA(18:0_20:4)<br>PA(20:2_18:2)	30
50	723.5607	DG(41:3)	[M+Cl]-	-0.0093	-12.85	723.57	Harmonic	n/a	n/a	n/a

51	725.5122	PA(38:3)	[M-H]-	-0.0005	-0.69	725.5127	Diathermy	YES	PA(18:0_20:3)	30
52	727.5272	PA(38:2)	[M-H]-	-0.0011	-1.51	727.5283	Diathermy	YES	PA(18:2_20:0)	30
53	728.5311	PE(35:2)	[M-H]-	0.0075	10.29	728.5236	Dathermy	YES	n/a	30
54	731.5432	DG(42:6)	[M+Cl]-	0.0045	6.15	731.5387	Harmonic	n/a	n/a	n/a
55	733.5612	DG(42:5)	[M+Cl]-	0.0069	9.41	733.5543	Harmonic	YES	n/a	n/a
56	737.5103	PA(39:4)	[M-H]-	-0.0024	-3.25	737.5127	Diathermy	n/a	n/a	n/a
57	738.513	PE(36:4)	[M-H]-	0.0051	6.91	738.5079	Diathermy	YES	PE(20:4_16:0)	35
58	742.5381	PE(36:2)	[M-H]-	-0.0011	-1.48	742.5392	Diathermy	n/a	n/a	n/a
59	744.5513	PE(36:1)	[M-H]-	-0.0036	-4.84	744.5549	Diathermy	YES	PE(18:1_18:0)	30-50
60	747.5109	PG(34:1)	[M-H]-	-0.0073	-9.77	747.5182	Diathermy	YES	PG(18:1_16:0)	35
61	749.5127	PA(40:5)	[M-H]-	0	0.00	749.5127	Diathermy	YES	PA(18:0_22:5)	35
62	750.5166	PE(37:5)	[M-H]-	0.0087	11.59	750.5079	Diathermy	YES	n/a	30
63	766.538	PE(38:4)	[M-H]-	-0.0012	-1.57	766.5392	Diathermy	YES	PE(18:0_20:4)	30-50
64	769.5088	PG(36:4)	[M-H]-	0.0063	8.19	769.5025	Diathermy	YES	PG(18:2_18:2)<br>PG(18:3_18:1)	30
65	770.5648	PE(38:2)	[M-H]-	-0.0057	-7.40	770.5705	Diathermy	YES	PE(18:1_20:1)<br>PE(18:2_20:0)<br>PE(22:2_16:0)	30-50
66	772.5723	DGCC(36:5)	[M-H]-	-0.001	-1.29	772.5733	Harmonic	n/a	n/a	n/a
67	773.5298	PG(36:2)	[M-H]-	-0.004	-5.17	773.5338	Diathermy	YES	PG(18:1_18:1)<br>PG(18:2_18:0)	30-50
68	775.5422	PG(36:1)	[M-H]-	-0.0073	-9.41	775.5495	Diathermy	YES	PG(18:0_18:1)	30-50
69	792.548	PE(40:5)	[M-H]-	-0.0069	-8.71	792.5549	Diathermy	YES	PE(18:0_22:5)	35
70	794.5675	PE(40:4)	[M-H]-	-0.003	-3.78	794.5705	Diathermy	YES	PE(22:4_18:0)	30-50
71	796.5781	PE(40:3)	[M-H]-	-0.0079	-9.92	796.5860[illegible]	Diathermy	YES	PE(20:3_18:0)	35
72	798.5942	PE(40:2)	[M-H]-	-0.0076	-9.52	798.6018	Harmonic	YES	PE(18:0_22:2)<br>PE(22:1_18:1)	35
73	816.5522	PE(42:7)	[M-H]-	-0.0027	-3.31	816.5549	Diathermy	YES	PE(20:1_22:6)	35
74	833.5513	PI(O-35:2)	[M-H]-	-0.0036	-4.32	833.5549	Diathermy	YES	PI(16:0_18:2)<br>PI(18:1_16:1)	35
75	850.5596	PS(41:5)	[M-H]-	-0.0008	-0.94	850.5604	Diathermy	YES	n/a	n/a
76	871.739	PE-Cer(t48:1)	[M-H]-	0.0116	13.31	871.7274	Harmonic	n/a	n/a	n/a
77	873.7545	PE-Cer(t48:0)	[M-H]-	0.0115	13.16	873.7430	Harmonic	n/a	n/a	n/a
78	885.5515	PI(38:4)	[M-H]-	0.0016	1.81	885.5499	Diathermy	YES	PI(20:4_18:0)	40
79	895.7508	TG(52:1)	[M+Cl]-	-0.0019	-2.12	895.7527	Harmonic	n/a	n/a	n/a
80	897.7664	TG(52:0)	[M+Cl]-	-0.0019	-2.12	897.7683	Harmonic	n/a	n/a	n/a
81	899.782	PE-Cer(d51:0)	[M-H]-	-0.0131	-14.56	899.7951	Harmonic	n/a	n/a	n/a
82	900.7809	CerP(t53:0)	[M-H]-	0.0018	2.00	900.7791	Harmonic	n/a	n/a	n/a
83	901.7854	TG(O-53:3)	[M-H]-	-0.0012	-1.33	901.7866	Harmonic	n/a	n/a	n/a
84	919.7517	TG(54:3)	[M+Cl]-	-0.001	-1.09	919.7527	Harmonic	n/a	n/a	n/a
85	921.7669	TG(54:2)	[M+Cl]-	-0.0014	-1.52	921.7683	Harmonic	n/a	n/a	n/a
86	923.7844	TG(54:1)	[M+Cl]-	0.0004	0.43	923.784	Harmonic	n/a	n/a	n/a
87	925.7992	TG(54:0)	[M+Cl]-	-0.0004	-0.43	925.7996	Harmonic	n/a	n/a	n/a
88	927.8007	TG(O-55:4)	[M+Formate]-	-0.0015	-1.62	927.8022	Harmonic	n/a	n/a	n/a
89	947.7856	TG(56:3)	[M+Cl]-	0.0016	1.69	947.784	Harmonic	n/a	n/a	n/a
90	949.7978	TG(56:2)	[M+Cl]-	-0.0018	-1.90	949.7996	Harmonic	n/a	n/a	n/a
91	971.7861	TG(58:5)	[M+Cl]-	0.0021	2.16	971.784	Harmonic	n/a	n/a	n/a
92	973.7999	TG(58:4)	[M+Cl]-	0.0003	0.31	973.7996	Harmonic	n/a	n/a	n/a
93	975.808	TG(58:3)	[M+Cl]-	-0.0073	-7.48	975.8153	Harmonic	n/a	n/a	n/a

Table Matrix 2: Tentative identifications and MSMS data in the *m/z* range of 600-1000 in positive mode

Tentative identifications and MSMS data of peaks in the *m/z* range of 600 to 1000 in positive mode. Accurate masses of candidate *m/z* peaks, to four decimal places after lock mass correction (Leucine Enkephalin at *m/z* 556.2771) were used to interrogate the LIPID MAPS database (www.lipidmaps.org) with the highest ranked match, based on delta value (< 0.05). A list is given below whether the *m/z* peak observed is more abundant in the Harmonic or diathermy REIMS data.(*DG-Diglyceride, Cer-Ceramide, SQDG-Sulfoquinovosyldiacylglycerol, LPS-Lipopolysaccharide, PA-Phosphatidic acid, PC-Phosphatidylcholine, PE-Phosphatidylethanolamine, HexCer-Hexosyl Ceramide, TG-Triglyceride)*

	m/z observed	ID	Adduct	Delta value	ppm error	m/z Theoretical	Higher abundance	Source	MSMS	Fatty acid composition	Collison energy (V)
1	603.5356	DG(O-36:4)	[M+H]+	-0.0009	-1.49	603.5347	diathermy	Lipid maps	yes	na	30-50
2	605.5509	DG(O-36:3)	[M+H]+	-0.0006	-0.99	605.5503	diathermy	Lipid maps	yes	na	30-50
3	617.5151	DG(36:4)	[M+H]+	-0.0012	-1.94	617.5139	diathermy	Lipid maps	yes	na	30-50
4	627.5352	DG(O-38:6)	[M+H]+	-0.0005	-0.80	627.5347	diathermy	Lipid maps	yes	na	30-50
5	630.6202	Cer(d42:2)	[M+H-H2O]+	-0.0019	-3.01	630.6183	diathermy	Lipid maps	yes	Cer(d18:1_24:1)	30-50
6	632.6356	Cer(d42:1)	[M+H-H2O]+	-0.0016	-2.53	632.634	harmonic	Lipid maps	yes	Cer(d18:1_24:0)	30-50
7	643.5285	DG(38:5)	[M+H]+	0.0011	1.71	643.5296	diathermy	Lipid maps	yes	na	30-50
8	645.5468	DG(38:4)	[M+H]+	-0.0016	-2.48	645.5452	diathermy	Lipid maps	yes	na	30-50
9	667.5249	DG(38:4)	[M+Na]+	0.0023	3.45	667.5272	diathermy	Lipid maps	yes	na	30-50
10	669.5438	DG(38:3)	[M+Na]+	-0.001	-1.49	669.5428	diathermy	Lipid maps	yes	na	30-50
11	671.5602	DG(40:5)	[M+H]+	0.0007	1.04	671.5609	diathermy	Lipid maps	yes	na	30-50
12	716.6196	DG(42:5)	[M+NH4]+	-0.0009	-1.26	716.6187	diathermy	Lipid maps	yes	na	30-50
13	737.4506	SQDG(28:1)	[M+H]+	-0.0002	-0.27	737.4504	diathermy	Lipid maps	yes	na	30-50
14	739.4664	SQDG(28:0)	[M+H]+	-0.0003	-0.41	739.4661	diathermy	Lipid maps	yes	na	30-50
15	740.4855	LPS(34:5)	[M+H]+	0.0006	0.81	740.4861	diathermy	Lipid maps	yes	na	30-50
16	751.5209	PA(38:2)	[M+Na]+	0.0039	5.19	751.5248	diathermy	Lipid maps	yes	na	30-50
17	756.5956	PC(35:1)	[M+H-H2O]+	-0.0054	-7.14	756.5902	harmonic	Lipid maps	yes	na	30-50
18	758.579	PC(34:2)<br>PE(37:2)	[M+H]+<br>[M+H]+	-0.0096	-12.66	758.5694	diathermy	Lipid maps	NO	na	30-50
19	760.5793	PC(34:1) ⍰	[M+H]+	0.0058	7.63	760.5851	diathermy	Lipid maps	yes	PC(16:0_18:1)	30-50
20	765.4835	SQDG(30:1)	[M+H]+	-0.0018	-2.35	765.4817	diathermy	Lipid maps	yes	na	30-50
21	768.5503	PE(38:4)	[M+H]+	0.0035	4.55	768.5538	diathermy	Lipid maps	yes	na	30-50
22	772.5844	PC(35:2)	[M+H]+	0.0007	0.91	772.5851	diathermy	Lipid maps	yes	na	30-50
23	774.598	PC(35:1)	[M+H]+	0.0027	3.49	774.6007	diathermy	Lipid maps	yes	na	30-50
24	782.5463	SHexCer(d34:0)	[M+H]+	-0.0016	-2.04	782.5447	diathermy	Lipid maps	yes	na	30-50
25	784.5496	PC(35:4(OH))	[M+H]+	-0.0009	-1.15	784.5487	diathermy	Lipid maps	yes	na	30-50
26	796.5806	PC(37:4)<br>PE(40:4)	[M+H]+<br>[M+H]+	0.0045	5.65	796.5851	diathermy	Lipid maps	no	na	30-50
27	806.5106	PC(35:4)	[M+K]+	-0.0009	-1.12	806.5097	diathermy	Lipid maps	yes	na	30-50
28	810.5785	PC(O-36:2) ⍰	[M+K]+	-0.0012	-1.48	810.5773	diathermy	Lipid maps	yes	na	30-50
29	812.5866	PC(37:4(OH))	[M+H]+	-0.0066	-8.12	812.58	diathermy	Lipid maps	yes	na	30-50
30	836.5853	PC(39:6(OH))	[M+H]+	-0.0053	-6.34	836.58	diathermy	Lipid maps	yes	na	30-50
31	838.62	PC(38:1)	[M+Na]+	0.0096	11.45	838.6296	diathermy	Lipid maps	yes	na	30-50
32	840.6111	PC(39:4(OH))	[M+H]+	0.0002	0.24	840.6113	diathermy	Lipid maps	yes	na	30-50
33	848.7731	TG(50:2)	[M+NH4]+	-0.0029	-3.42	848.7702	harmonic	Lipid maps	yes	TG(16:0_18:2_16:0)	30-50
34	862.5907	PC(41:7(OH))	[M+H]+	0.0049	5.68	862.5956	diathermy	Lipid maps	yes	na	30-50
35	864.6053	PC(39:3(OH))	[M+Na]+	0.0036	4.16	864.6089	diathermy	Lipid maps	yes	na	30-50
36	874.7897	TG(52:3)	[M+NH4]+	-0.0039	-4.46	874.7858	harmonic	Lipid maps	yes	TG(18:0_18:2_16:0)	30-50
37	876.8058	TG(52:2)	[M+NH4]+	-0.0043	-4.90	876.8015	harmonic	Lipid maps	yes	TG(18:1_18:1_16:0)	30-50
38	902.8212	TG(54:3)	[M+NH4]+	-0.0041	-4.54	902.8171	harmonic	Lipid maps	yes	TG(18:1_18:0_18:2)	30-50
39	904.8358	TG(54:2)	[M+NH4]+	-0.003	-3.32	904.8328	harmonic	Lipid maps	yes	TG(18:1_18:1_18:0)	30-50
40	916.8267	TG(55:3)	[M+NH4]+	0.0061	6.65	916.8328	harmonic	Lipid maps	yes	na	30-50
41	918.8367	TG(55:2)	[M+NH4]+	0.0117	12.73	918.8484	harmonic	Lipid maps	yes	na	30-50
42	928.8311	TG(56:4)	[M+NH4]+	0.0017	1.83	928.8328	harmonic	Lipid maps	yes	na	30-50
43	930.8499	TG(56:3)	[M+NH4]+	-0.0015	-1.61	930.8484	harmonic	Lipid maps		na	30-50
44	969.8431	PC(O-49:3)	[M+NH4]+	-0.0073	-7.53	969.8358	harmonic	Lipid maps	yes	na	30-50
45	973.8708	TG(62:5)	[M+H-H2O]+	-0.012	-12.32	973.8588	harmonic	Lipid maps	yes	na	30-50
46	983.8755	TG(O-59:1)	[M+K]+	0.0012	1.22	983.8767	harmonic	Lipid maps	yes	na	30-50
